# Evaluation of markers of immunity in different metastatic immune microenvironments suggests more suppression within breast to liver metastases in breast cancer

**DOI:** 10.1007/s10549-024-07295-w

**Published:** 2024-04-20

**Authors:** Robert Hsu, Batul Al-zubeidy, Daniel Flores, Ari Nazarian, Aaron Baugh, Edgar Gonzalez, Sofi Castanon, Joanne Xiu, Irene Kang, Darcy Spicer, Heinz Josef Lenz, Lily Dara, Foluso O. Ademuyiwa, W. Michael Korn, Sheeba Irshad, Isaac S. Chan, Evanthia T. Roussos Torres

**Affiliations:** 1 Division of Medical Oncology, Department of Medicine, University of Southern California Norris Comprehensive Cancer Center, Keck School of Medicine, University of Southern California, Los Angeles, CA USA; 2 Department of Medicine, Keck School of Medicine, University of Southern California, Los Angeles, CA USA; 3 Caris Life Sciences, Phoenix, AZ USA; 4 Present Address: Department of Medical Oncology & Therapeutics Research, City of Hope Orange County, Irvine, CA USA; 5 Division of Gastrointestinal and Liver Diseases, Department of Medicine, Keck School of Medicine, University of Southern California, Los Angeles, CA USA; 6 Washington University School of Medicine, St. Louis, MO USA; 7 School of Cancer & Pharmaceutical Sciences, King’s College London, Cancer Research UK (CRUK) Clinician Scientist, London, UK; 8 Division of Hematology and Oncology, Department of Internal Medicine, University of Texas Southwestern, Dallas, TX USA; 9 Harold C. Simmons Comprehensive Cancer Center, University of Texas Southwestern Medical Center, Dallas, TX USA

**Keywords:** Metastatic breast cancer, Liver metastasis, Tumor microenvironment, Tumor immune infiltration

## Abstract

**Purpose:**

Programmed death receptor ligand-1 (PD-L1) expression and tumor mutational burden (TMB) are approved screening biomarkers for immune checkpoint inhibition (ICI) in advanced triple negative breast cancer. We examined these biomarkers along with characterization of the tumor microenvironment (TME) between breast tumors (BrTs), axillary metastases (AxMs), liver metastases (LvMs), non-axillary lymph node metastases, and non-liver metastases to determine differences related to site of metastatic disease.

**Methods:**

3076 unpaired biopsies from breast cancer patients were analyzed using whole transcriptome sequencing and NextGen DNA depicting TMB within tumor sites. The PD-L1 positivity was determined with VENTANA PD-L1 (SP142) assay. The immune cell fraction within the TME was calculated by QuantiSeq and MCP-counter.

**Results:**

Compared to BrT, more LvM samples had a high TMB (≥ 10 mutations/Mb) and fewer LvM samples had PD-L1^+^ expression. Evaluation of the TME revealed that LvM sites harbored lower infiltration of adaptive immune cells, such as CD4^+^, CD8^+^, and regulatory T-cells compared with the BrT foci. We saw differences in innate immune cell infiltration in LvM compared to BrT, including neutrophils and NK cells.

**Conclusions:**

LvMs are less likely to express PD-L1^+^ tumor cells but more likely to harbor high TMB as compared to BrTs. Unlike AxMs, LvMs represent a more immunosuppressed TME and demonstrate lower gene expression associated with adaptive immunity compared to BrTs. These findings suggest biopsy site be considered when interpreting results that influence ICI use for treatment and further investigation of immune composition and biomarkers expression by metastatic site.

**Supplementary Information:**

The online version contains supplementary material available at 10.1007/s10549-024-07295-w.

## Introduction

Inhibition of immune checkpoint regulators, such as programmed cell death protein-1 (PD-1) and its receptor PD-L1 have improved cancer treatment outcomes [1]. While PD-L1 expression and tumor mutational burden (TMB) have been used as predictive biomarkers of efficacy to immune checkpoint inhibitors (ICI), their expression is often discordant with response [2, 3]. Recent meta-analysis of pooled PD-L1 in patients with breast cancer of different histologies demonstrated that only 24% of tumor, 33% of immune, and 25% of both immune and tumor cells expressed PD-L1 [1, 4]. The highest PD-L1 expression was seen in triple negative breast cancers (TNBC), in patients with higher pathological complete response to neoadjuvant chemoimmunotherapy [5]. In addition, response to ICIs in patients with metastatic TNBC was found to correlate with PD-L1 positivity (PD-L1^+^) [6]^.^ However, there are numerous studies in which PD-L1 expression did not correlate with response along with discordance in PD-L1 expression between the primary and metastatic biopsy site, and there is little guidance about consideration of PD-L1 expression and TMB with regard to tumor biopsy site in the metastatic setting [7–10]^.^

Immune cell topography at different metastatic sites can serve as an alternative paradigm to inform response. Immune TME profiling of breast tumor metastases using single-cell RNA sequencing relative to primary breast tumors revealed greater immune-suppression with T-cell exhaustion, clonal expansion of regulatory T-cells (T-regs) and increased M2-like tumor associated macrophages (TAM) corelating with worse outcomes [11–13]. Tumor heterogeneity, divergent immune and tumor cell expression of PD-L1, and diverse immunohistochemistry (IHC) assays and scoring methods make sole use of PD-L1^+^ unreliable as a predictive biomarker of response [14–16]. Meta-analysis evaluating the discordance rate of PD-L1^+^ showed 51.2% of immune cells within primary sites stained positive vs. 37.1% of metastatic foci, and 30.1% of tumor cells in primary sites vs. 14.6% in distant sites and on further analysis of studies with matched primary and metastatic biopsies 13.6% of patients had discordance when PD-L1 status was assessed on tumor cells, 39.5% when assessed on immune cells, and 47.6% when assessed on both tumor and immune cells [10]. Studies investigating the clinical validity and utility of tumor infiltrating lymphocyte count (CD8^+^, CD4^+^, T-helper, and dendritic cells) and PD-L1 expression as therapeutic biomarkers demonstrated their decreased infiltration in metastatic sites compared with primary lesions, which perhaps contribute to underlying ICI resistance [17, 18].

We queried 3076 unpaired breast cancer tumors consisting of biopsies grouped by anatomic location: breast (BrT), axillary metastasis (AxM), liver metastasis (LvM), non-axillary lymph node metastasis (NAxLNM), and non-liver non-lymph node metastasis (NLvM). The primary objectives were to determine differences in PD-L1^+^ and TMB status based on site of biopsy and identify possible immune cell targets differentially expressed in immune cells by biopsy site, particularly in LvM. Previous studies show that the abundance of adaptive immune cells is reduced at distant metastatic sites compared to primary sites [19, 20]. There have been mixed results about differences in abundance of macrophages at distant metastatic sites and reported decrease of T-regs [19, 20]. However, it has been clearest amongst previous studies of a differing TME in metastatic breast cancer sites [19, 20]. We focused our TME analysis on LvM given that up to 50% of patients with metastatic breast cancer present with LvM, often with very poor overall survival rates of 4–8 months [21, 22]. It is well studied that the liver is an immune suppressed organ and thus consideration of the immune cell components of the liver and other sites of distant metastasis should be considered when investigating disease progression or response to ICIs [23–25]

## Methods

### Study cohort

Tumor samples that underwent comprehensive molecular profiling at Caris Life Sciences (Phoenix, AZ) were retrospectively investigated for immune-related molecular features. This study was conducted in accordance with guidelines of the Declaration of Helsinki, Belmont report, and U.S. Common rule. In keeping with 45 CFR 46.101(b) [1], this study was performed utilizing retrospective, deidentified clinical data. Therefore, this study was considered IRB exempt and no patient consent was necessary.

### Next generation sequencing (NGS)

NGS was performed on genomic DNA isolated from formalin-fixed paraffin-embedded (FFPE) samples using the NextSeq platform (Illumina, Inc., San Diego, CA). Matched normal tissue was not sequenced. A custom-designed SureSelect XT assay was used to enrich 592 whole-gene targets (Agilent Technologies, Santa Clara, CA). All variants were detected with > 99% confidence based on allele frequency and amplicon coverage, with an average sequencing depth of coverage of > 500 and an analytic sensitivity of 5%. Prior to molecular testing, tumor enrichment was achieved by harvesting targeted tissue using manual microdissection techniques. Genetic variants identified were interpreted by board-certified molecular geneticists and categorized as ‘pathogenic’, ‘likely pathogenic’, ‘variant of unknown significance’, ‘likely benign’, or ‘benign’, according to the American College of Medical Genetics and Genomics (ACMG) standards. When assessing mutation frequencies of individual genes,’pathogenic’ and ‘likely pathogenic’ were counted as mutations while others excluded [23].

### TMB

TMB included all non-synonymous missense, nonsense, inframe insertion/deletion and frameshift mutations per tumor not previously described as germline alterations in dbSNP151, Genome Aggregation Database (gnomAD) or benign variants identified by Caris geneticists. A cutoff point of ≥ 10 mutations per MB was used based on the KEYNOTE-158 pembrolizumab trial [24].

### RNA expression method

FFPE specimens were scrutinized to contain a minimum of 10% tumor content for enrichment and extraction of tumor-specific RNA. Qiagen RNA FFPE tissue extraction kit was used, and the RNA quality and quantity determined via Agilent TapeStation. Biotinylated RNA baits were hybridized to the synthesized and purified cDNA targets and the bait-target complexes were amplified in a post capture PCR reaction. The Illumina NovaSeq 6500 was used to sequence the whole transcriptome from patients to an average of 60 M reads. Raw data were demultiplexed by Illumina Dragen BioIT accelerator, trimmed, counted, PCR-duplicates removed and aligned to human reference genome hg19 by STAR aligner. For transcription counting, transcripts per million (TPM) was generated using the Salmon expression pipeline. Immune cell fraction was calculated by QuantiSeq and Microenvironment Cell Populations-counter (MCP-counter) [23, 25, 26]. Previously established gene signatures to evaluate M1 and M2 macrophages, myeloid derived suppressor cells (MDSCs) and regulatory T-cells (T-regs) [23, 27]. We also curated gene lists representative of checkpoint inhibition and stimulation [28, 29]. For specific investigation of certain immune cell populations, we used previously established gene signatures to evaluate M1 macrophages, M2 macrophages, and T-regs [27].

### IHC analysis

Slides were stained using automated staining techniques and optimized and validated per Clinical Laboratory Improvement Amendments (CLIA)/Clinical Outcome Assessment (COA) and International Organization for Standardization (ISO) requirements. The VENTANA PD-L1 (SP142) assay was used to score PD-L1^+^on immune cells with staining $\ge$ 1% was considered positive. Of note, these studies were conducted prior to the standardization of CPS scores.

### Inflamed T-cell analysis

Tumors were categorized into non-T-cell inflamed, T-cell inflamed, and intermediate using defined T-cell inflamed expression signature consisting of 160 genes [30]. The normalized expression was transformed into a scoring system in which each gene is defined as upregulated (+ 1), downregulated (−1), or unchanged (0) relative to the mean. Scores of all genes ranged from -160 to 160. Scores ≤ 80 were categorized as non-T-cell inflamed while scores > 80 were categorized as T-cell inflamed, and the rest were intermediate [30].

### Statistical analysis

Percentage of tumors with TMB $\ge$ 10 Mb/mutation and PD-L1^+^ were analyzed using Fisher Exact tests. TME cell fractions were analyzed using QuantiSeq and MCP-counter. Continuous variables were compared using non-parametric tests including Wilcoxon/Mann Whitney-*U* tests. *p*-values with multiple comparisons were further corrected using Benjamini–Hochberg method to avoid type-I error and an adjusted *p*-value (*q*-value) of < 0.05 was considered a significant difference.

## Results

### Tumor characteristics

Females made up 99.1% with median age of 60 years (Table 1a). Hormone receptor positive and human epidermal growth factor receptor 2 negative (HR^+^/HER2^−^) was the most common clinical subtype (55.6%) followed by TNBC (27.7%), and HER2+ (8.0%) (Table 1b). In LvMs, 69.5% of tumors were HR^+^/HER2^−^ (Table 1b). There were 1274 BrTs, 291 AxMs, 495 LvMs, 124 NAxLNMs, and 892 NLvMs. Within NLvM, bone (22.2%), lung (14.8%), and chest wall (13.0%) were the most common metastatic sites, respectively (Table 1c).

**Table 1:** A—Patient characteristics broken down by biopsy site, B—location of biopsies by subtype, C—location of non-liver/non-axilla specimen sites

A							
		Breast (BrT) (*n* = 1274) (%)	Axillary (AxM) (*n* = 291) (%)	Liver (LvM) (*n* = 495) (%)	Non-axillary lymph node (NAxLNM) (*n* = 124) (%)	Non-liver Met (NLvM) (*n* = 892) (%)	Total (*n* = 3076) (%)
Gender	Female	1264 (99.2)	284 (97.6)	494 (99.8)	123 (99.1)	882 (98.9)	3047 (99.1)
	Male	10 (0.8)	7 (2.4)	1 (0.2)	1 (0.9)	10 (1.1)	29 (0.9)
Age (Years)	Range	22–94	28–89	24–92	28–88	27–93	22–93
	Median	58	61	61	60.5	60	60

B					
	Her2^+^ (*n* = 247) (%)	HR^+^ Her2^−^ (*n* = 1712) (%)	TNBC (*n* = 853) (%)	Unclear (*n* = 264) (%)	Total
Breast (BrT)	107 (43.3)	638 (37.3)	427 (50.1)	102 (38.6)	1274
Axillary (AxM)	19 (7.7)	152 (8.9)	102 (12.0)	18 (6.8)	291
Liver (LvM)	27 (10.9)	344 (20.1)	71 (8.3)	53 (20.1)	495
Non-Axillary Lymph Node (NAxLNM)	8 (6.5)	61 (49.2)	46 (37.1)	9 (7.3)	124
Non-liver Met (NLvM)	94 (38.1)	578 (33.8)	253 (29.7)	91 (34.5)	892
Total	247 (8.0)	1712 (55.6)	853 (27.7)	264 (8.6)	3076

C	
Non-liver/non-axilla specimen sites	*N* = 892 (%)
Bone	198 (22.2)
Brain	93 (10.4)
Chest/chest wall	116 (13.0)
Connective tissue	43 (4.8)
GI organ	97 (10.9)
GYN organs	31 (3.5)
Lung	132 (14.8)
Other	81 (9.1)
Pleura	69 (7.7)
Skin	32 (3.6)

### Higher frequency of TMB-high tumors observed in distant metastases

**Fig. 1:**
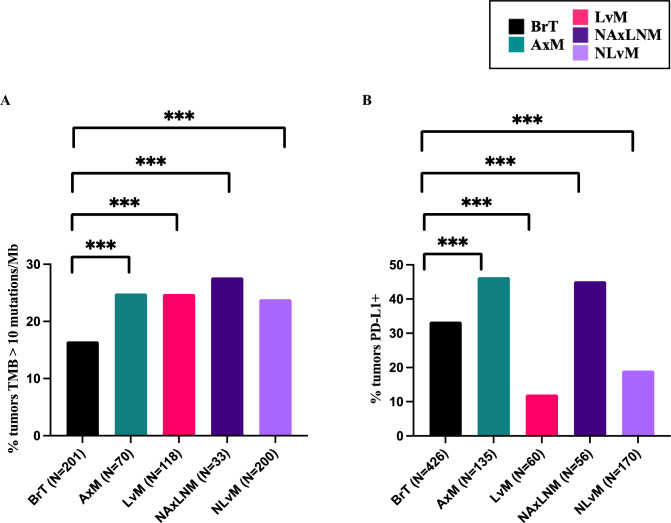
**A** Percent tumors with TMB $\ge$ 10 Mutations/Mb by biopsy site for all tumors, **B** percent tumors with PD-L1^+^ for all tumors

TMB varied across sites with the highest variance seen in LvMs ranging from 2 and 99 mutations/Mb, followed by NLvMs (Supplemental Table 1). We noted significantly higher percentage of tumors classified as TMB-high ($\ge$ 10 mutations/Mb) in all distant sites as compared to BrTs (16.5%); AxMs (24.9%, *q*-value = 0.0013), LvMs (24.8%, *q*-value = 0.0002), NAxLNMs (27.7%, *q*-value = 0.0021), and NLvMs (23.9%, *q*-value < 0.0001) (Fig. 1a, Supplemental Fig. 2a). These data suggest that distant metastatic sites are likely to be classified as TMB-High compared with primary tumors.

### PD-L1 expression varied greatly by site of tumor growth- lowest in liver metastases and highest in the axilla

With regards to PD-L1^+^, LvMs has the lowest percentage of PD-L1^+^ tumors (12.1%) compared with BrTs (33.4%, *q*-value < 0.0001) while AxMs had the highest percentage of PD-L1^+^ tumors (46.4%) (Fig. 1b, Supplemental Table 2b). These results suggest that AxMs are more likely to harbor PD-L1^+^ tumor cells than the BrT, while LvMs are least likely.

### Evaluation of immune cell composition using RNAseq suggests macrophage and T-cell infiltration vary by site

**Fig. 2:**
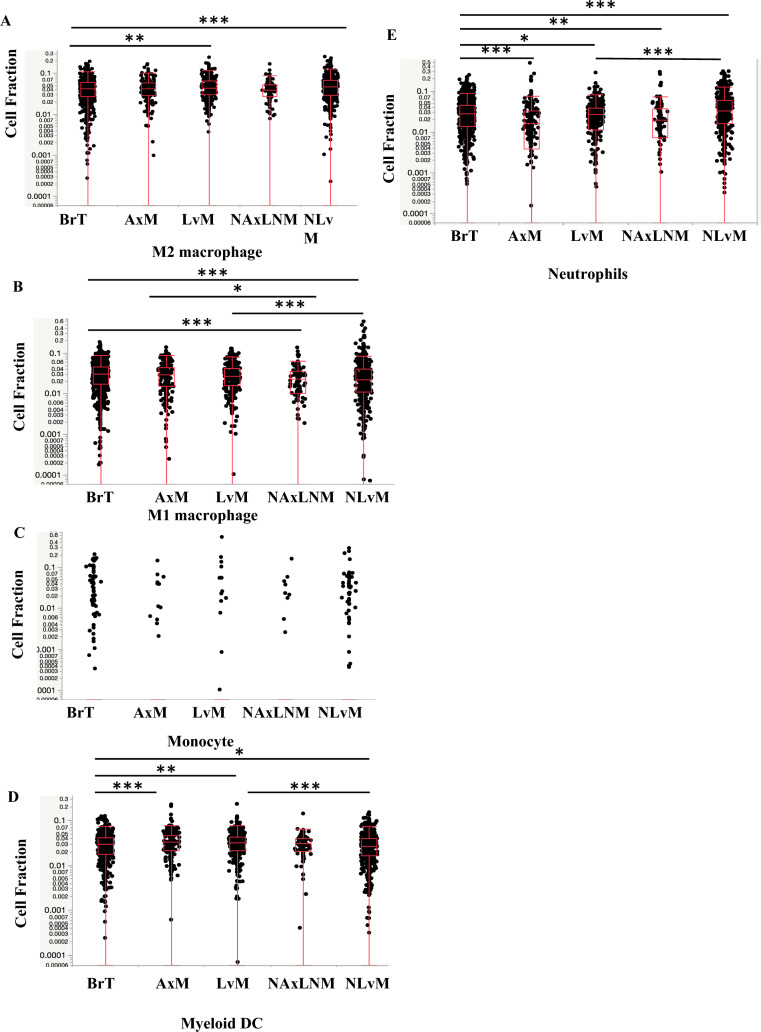
Expression of **A** M2 Macrophages, **B** M1 Macrophages, **C**. Monocytes, **D** Myeloid Dendritic Cells (DCs), **E**. Neutrophils by biopsy site measured in Cell Fraction using Quanti-Seq Method *q-value < 0.05, ** < 0.01, *** < 0.001

Next, we investigated bulk RNA sequencing data from tumor samples using two computational methods QuantiSeq, and MCP-Counter, to quantify different immune cell fractions as a surrogate for differences in immune cell composition. Compared to BrTs, LvMs have more M2-like TAMs, defined as anti-inflammatory macrophages which exert an immunosuppressive phenotype favoring tumor progression (Fig. 2a, Table 2a). LvMs showed no difference in M1-like anti-tumor macrophages and monocytes (Fig. 2b, c, Table 2a) but did show significantly more myeloid dendritic cells which typically promote an anti-tumor response, (Fig. 2d, Table 2a, b). The two quantification methods showed opposite trends for neutrophils and as such results are inconclusive (Figs. 2e, 4c, Table 2a, b).

**Table 2:** A—tumor microenvironment comparison of immune cell composition by biopsy site for all tumors using Quanti-Seq method. Significant increases noted in green and decreases noted in orange, B—tumor microenvironment comparison of immune cell composition by biopsy site for all tumors using MCP-counter method

A												
QuantiSeq Method	LvM relative to BrT	*q*-value	NLvM relative to BrT	*q*-value	AxM relative to BrT	*q*-value	NAxLNM relative to BrT	*q*-value	NLvM relative to LvM	*q*-value	NAxLNM relative to AxM	*q*-value
M1 macrophages		NS	*Decreased*	< 0.0001		NS	*Decreased*	< 0.0001	*Decreased*	< 0.0001	*Decreased*	0.0122
M2 macrophages	**Increased**	0.00225	**Increased**	< 0.0001		NS		NS		NS		NS
Monocytes		NS		NS		NS		NS		NS		NS
Myeloid dendritic cells	**Increased**	0.00675	*Decreased*	0.03400	**Increased**	0.00067		NS	*Decreased*	< 0.0001		NS
CD4 + T-cells	*Decreased*	< 0.0001	*Decreased*	0.04633	**Increased**	< 0.0001	**Increased**	< 0.0001	**Increased**	< 0.0001		NS
CD8 + T-cells	*Decreased*	< 0.0001	*Decreased*	< 0.0001	**Increased**	< 0.0001	**Increased**	0.0001	**Increased**	< 0.0001		NS
Regulatory T-cells	*Decreased*	< 0.0001	*Decreased*	0.02989	**Increased**	< 0.0001	**Increased**	< 0.0001	**Increased**	< 0.0001		NS
B-cells		NS		NS	**Increased**	< 0.0001	**Increased**	< 0.0001		NS		NS
Neutrophils	*Decreased*	0.0175	**Increased**	< 0.0001	*Decreased*	< 0.0001	*Decreased*	0.002	**Increased**	< 0.0001		NS
NK cells	**Increased**	0.01271	*Decreased*	0.00817	*Decreased*	< 0.0001	*Decreased*	0.0010	*Decreased*	< 0.0001		NS

B												
MCP-counter Method	LvM relative to BrT	*q*-value	NLvM relative to BrT	*q*-value	AxM relative to BrT	*q*-value	NAxLNM relative to BrT	*q*-value	NLvM relative to LvM	*q*-value	NAxLNM relative to AxM	*q*-value
Endothelial cells	*Decreased*	< 0.0001		NS		NS	*Decreased*	0.004	*Decreased*	< 0.0001	*Decreased*	0.015
Fibroblasts	*Decreased*	< 0.0001	*Decreased*	< 0.0001	*Decreased*	< 0.0001	*Decreased*	< 0.0001	**Increased**	< 0.0001	*Decreased*	< 0.0001
Monocytic lineage		NS	*Decreased*	< 0.0001	**Increased**	< 0.0001		NS		NS	*Decreased*	0.0006
Myeloid dendritic cells	*Decreased*	< 0.0001	*Decreased*	0.00057	**Increased**	0.0005		NS	**Increased**	< 0.0001	*Decreased*	0.0098
T-cells	*Decreased*	< 0.0001	*Decreased*	< 0.0001	**Increased**	< 0.0001	**Increased**	< 0.0001	**Increased**	< 0.0001		NS
CD8 + T-cells	*Decreased*	< 0.0001	*Decreased*	< 0.0001	**Increased**	< 0.0001	**Increased**	0.000167	**Increased**	< 0.0001		NS
Cytotoxic lymphocytes	*Decreased*	< 0.0001	*Decreased*	0.0040	**Increased**	< 0.0001	**Increased**	0.01722	**Increased**	< 0.0001		NS
B lineage	*Decreased*	< 0.0001	*Decreased*	< 0.0001	**Increased**	< 0.0001	**Increased**	< 0.0001		NS		NS
Neutrophils	**Increased**	< 0.0001	**Increased**	< 0.0001		NS		NS		NS		NS
NK cells	*Decreased*	0.00733		NS		NS		NS	Increased	< 0.0001		NS

Significant increases noted in bold and decreases noted in italic

**Fig. 3:**
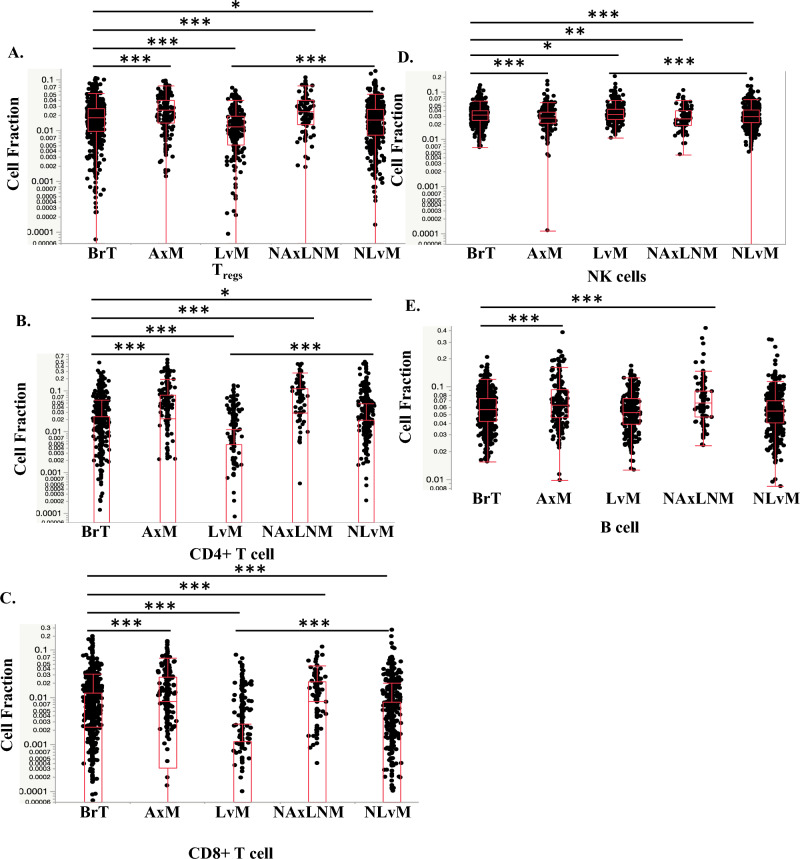
Expression of **A** T regulatory cells (Tregs), **B** CD4+ T-cells, **C** CD8+ T-cells, **D** Natural Killer (NK) cells. **E** B-cells by biopsy site measured in Cell Fraction using Quanti-Seq Method *q-value < 0.05, ** < 0.01, *** < 0.001

Evaluation of immunosuppressive lymphocytes reveal decreased T-regs by Quanti-Seq in LvM; T-regs were not calculated in MCP-Counter. Importantly, anti-tumor lymphocytes, CD4^+^ and CD8^+^ T-cells, were decreased in LvMs and NLvMs compared to BrT but increased in AxMs and NAxLNMs (Figs. 3c, 3b, 5a, Table 2). NK cells show opposite trends via the two quantification methods (Figs. 3d, 5b), while B-cells decreased by MCP-Counter only (Figs. 3e, 5c). In summary, NLvMs demonstrate a high level of suppressive immune cell composition as compared to BrTs while AxMs seem to harbor immune cells suggestive of a more immune-responsive environment.

**Fig. 4:**
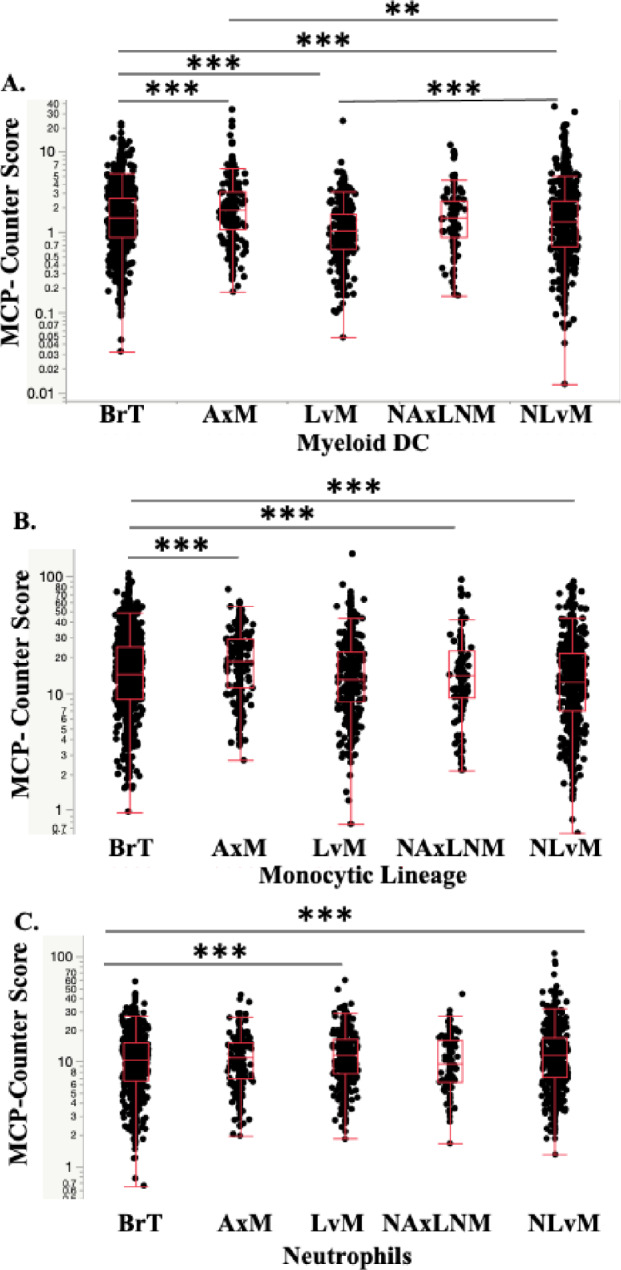
Expression of **A** Myeloid DCs, **B** Monocytes, **C** Neutrophils, by Biopsy Site using MCP-Counter Method *q-value < 0.05, ** < 0.01, *** < 0.001

**Fig. 5:**
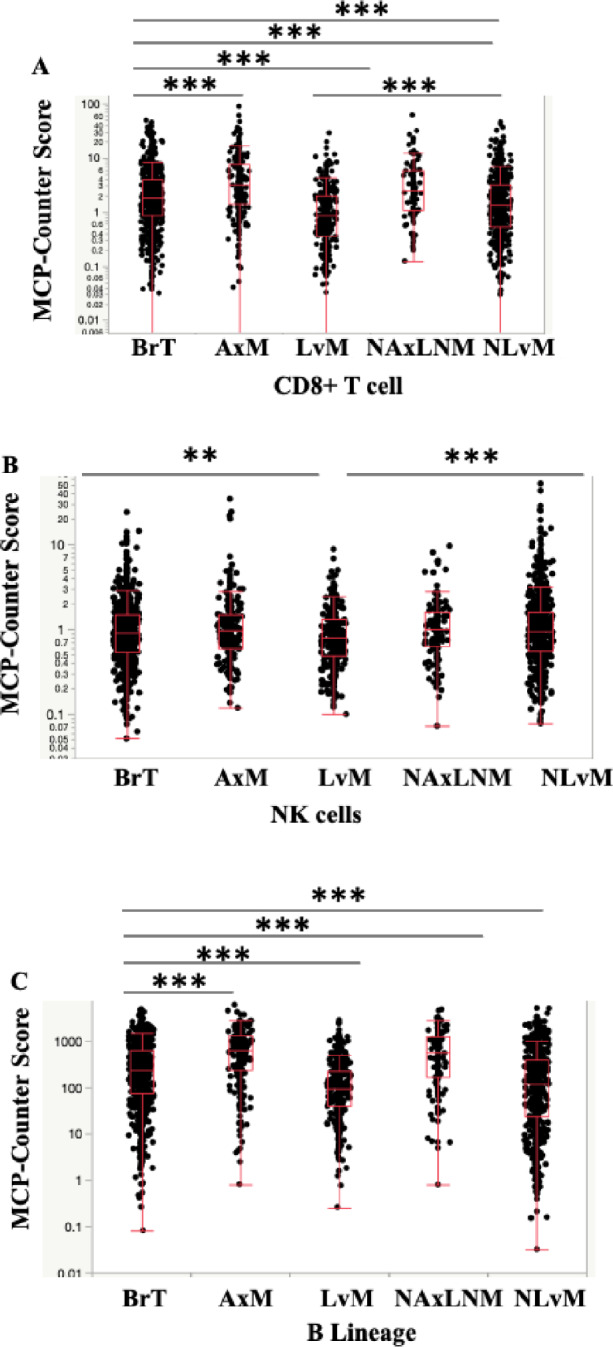
Expression of **A** CD8^+^ T-cells, **B** NK cells, **C** B cell lineage, by Biopsy Site Measured in Cell Fraction using MCP-Counter method *q-value < 0.05, ** < 0.01, *** < 0.001

**Fig. 6:**
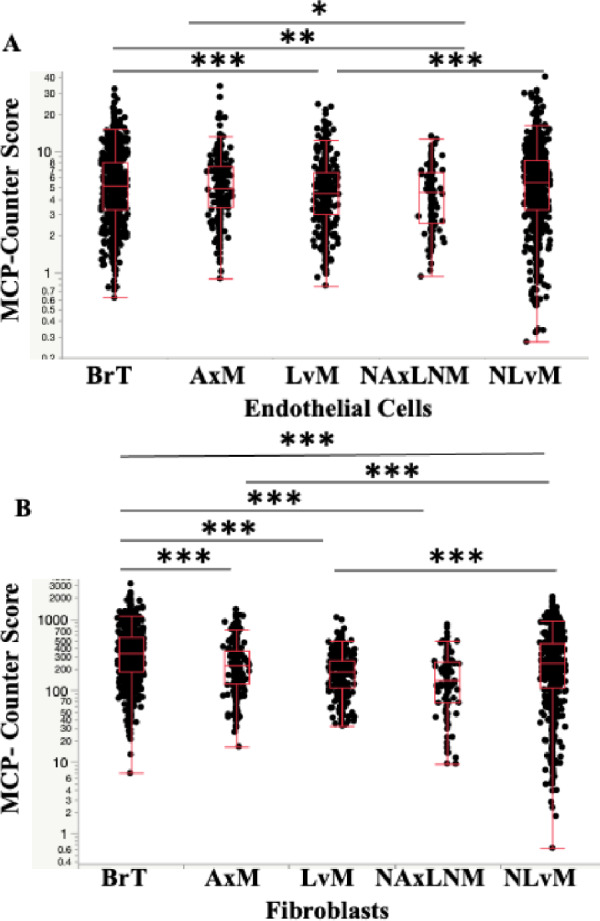
Expression of **A** Endothelial cells, **B** Fibroblasts, by Biopsy Site Measured in Cell Fraction using MCP-Counter method *q-value < 0.05, ** < 0.01, *** < 0.001

Investigation of cell fractions between metastatic sites showed that as compared to LvMs, NLvMs show fewer M1-like macrophages, DCs, T and NK cells and higher M2-like TAMs by QuantiSeq (Figs. 2, 3, 4, 5, 6, Table 2). Alternatively, AxMs show increases in T-regs but also CD4+ T-cells and CD8+ T-cells (Figs. 2, 3, 4, 5, 6, Table 2).

### Gene set enrichment analysis of immune cell subsets highlights varied genes of interest by metastatic site

**Fig. 7:**
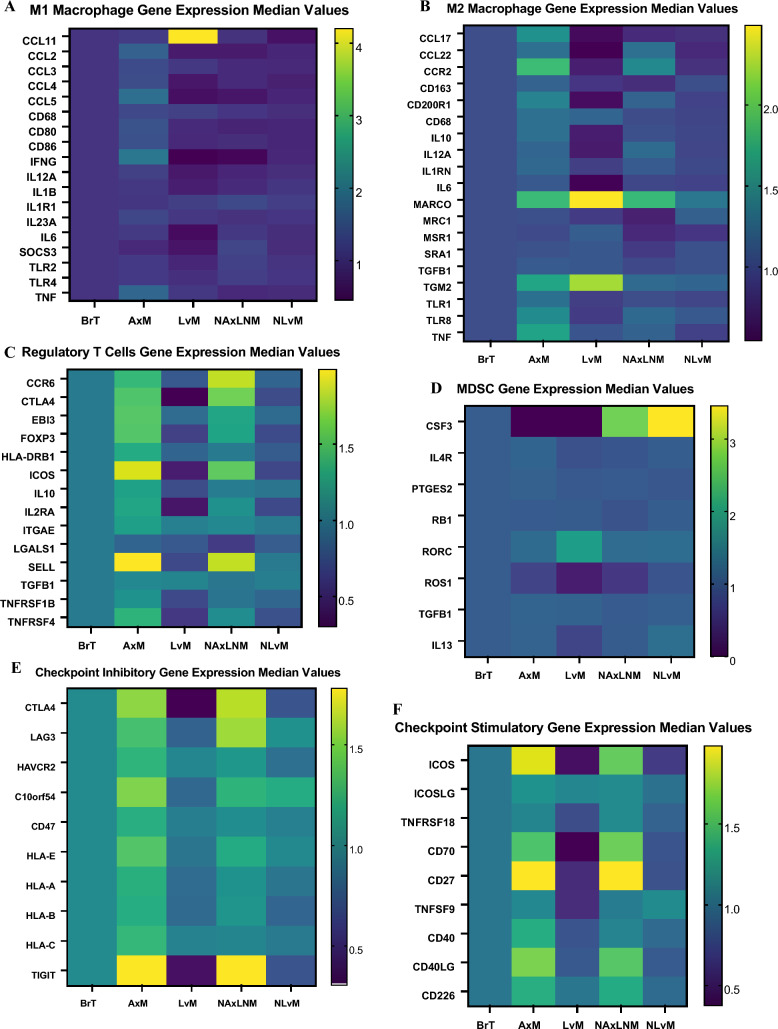
Heat maps demonstrating median value of gene expression of **A** M1 macrophage genes **B** M2 macrophage genes **C** Treg genes **D** MDSC genes **E** Checkpoint inhibitory genes **F** Checkpoint stimulatory genes in comparison to BrT values normalized to 1

To identify potential genes driving observed changes in immune cell composition by site, we examined gene expression in certain immune cell subsets. We found increased expression of inflammatory pathway genes including CCL4, CCL5, IFN-gamma, IL-12A, IL-1B, IL-6, SOCS3 in M1 macrophages from BrT compared to LvM, while there is increased expression of M2-polarizing CCL2, CD68, and NOS2 in M1 macrophages from LvM relative to BrT (Fig. 7a, Supplemental Table 3). In M2 macrophages, we noted increased expression of genes mediating inflammatory cytokines including IL-10, IL-12A, IL-6, TGM2, TLR-8 and others in BrT compared to LvM (Fig. 7b, Supplemental Table 4).

Investigation of T-reg genes showed that nearly all 14 genes had lower expression in LvM compared to BrT but higher expression in AxM (Fig. 7c; Supplemental Table 5). MDSC genes exhibited reduced expression of CSF3, IL4R, IL13, ROS1 and higher RORC, TGFB1, NOS2 expression in LvM relative to BrT (Fig. 7d; Supplemental Table 6).

We then investigated expression of checkpoint receptors and their ligands [29]. We show that most genes encoding checkpoints inhibiting immune response (CTLA4, LAG3, C10orf54, HLA-E, HLA-A, HLA-B, and TIGIT) and genes encoding checkpoints to promote immune response (ICOS, TNFRSF18, CD70, CD27, TNFSF9, CD40, and CD40LG) have significantly decreased expression in LvMs as compared to BrTs (*q*-value < 0.0001, < 0.001, respectively) and NLvMs (Fig. 7e, f; Supplemental Table 7). However, AxMs and NAxLNMs have higher gene expression of most checkpoints compared to BrTs including CTLA-4, LAG3, TIGIT and others (Fig. 7e, f; Supplemental Table 7). LvMs demonstrate lower gene expression of checkpoint inhibitors while AxMs exhibit increased immune up-regulating genes compared to BrT.

### Liver metastases demonstrates the lowest percentage of inflamed T-cells

**Fig.8:**
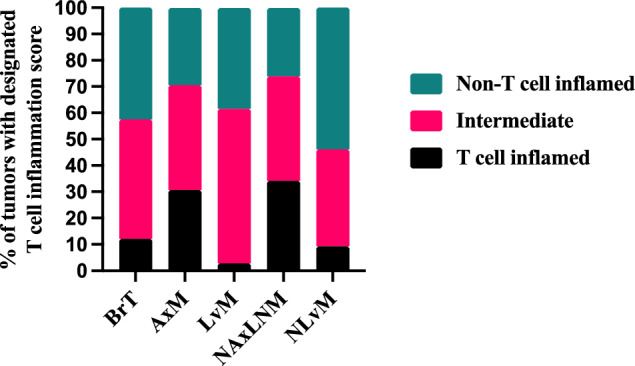
Percentage of T-cell inflamed, Intermediate, and non-T-cell Inflamed tumors across Biopsy Sites. *q-value < 0.05, ** < 0.01, *** < 0.001

An exploratory analysis using a published T-cell inflamed expression signature of 160 genes, revealed LvMs have a significantly smaller percentage of tumors with a T-cell inflamed signature (2.8%) compared to BrTs (12.8%; *q*-value < 0.0001) while NLvMs have a similar percentage (12.3%) [30]. However, NAxLNM (34.1%) and AxMs (30.7%) have a higher percentage of tumors with a T-cell inflamed signature as compared to samples from all other sites (Fig. 8; Supplemental Table 8).

### Analysis of data by breast cancer subtype reveals similar findings observed

We observed higher TMB across all distant biopsy sites compared to BrT in HR+ /HER2− but not in HER-2^+^ or TNBC (Supplemental Fig. 1b, Supplemental Table 2a). Similarly, we noted a similar pattern of decreased PD-L1^+^ tumor cells in LvM compared to BrT across all subtypes: HER-2^+^ (14.8% vs. 43.9%, *q*-value = 0.0110, HR^+^/HER-2^−^ (11.0% vs. 22.1%, *q*-value < 0.0001), TNBC (18.3% vs. 48.5%, *q*-value < 0.0001) (Supplemental Fig. 2, Supplemental Table 2b).

Regarding TME analysis of LvM as compared to BrTs by subtype using Quanti-Seq method, the only trends that were carried over from our combined analysis were the significant decreases seen in the T-cell subsets (T-regs, CD4^+^ and CD8^+^ T-cells) in LvMs which was observed in all subtypes. HR+ /HER2− demonstrated significantly more myeloid DCs. TNBC demonstrated significantly more NK cells (Supplemental Table 9b, c) which were also seen in the combined cohort in LvMs vs. BrTs. We see similar findings using the MCP-counter method whereby in LvM, there are significantly decreased abundance of T-cells, CD8^+^ T-cells, cytotoxic lymphocytes, B-cells, stromal fibroblasts and myeloid DCs in all subtypes (Supplemental Table 10a–d). Thus, a great majority of comparisons by subtype primarily in the HR^+^/HER-2^−^ and TNBC subtypes mirror findings in the total population and most non-significant comparisons are likely secondary to small sample size. Comparisons within subtypes between other metastatic sites can be found in Supplemental Tables 9 and 10.

## Discussion

Consistent with previous studies, our results show that TMB is higher in metastatic sites compared to BrTs, which may be a result of tumor clonal selection favoring features (“bottlenecking”) that are advantageous in distant sites [31]. We are one of the first to show that TMB level varies between different metastatic sites. LvM had the highest percentage of TMB-High tumors followed by NLvMs, AxMs then NAxLNMs. Evaluation of TMB from different metastatic sites matched by patient will be an important analysis to better understand if these differences are secondary to acquired features of the metastatic tumor cells themselves or if mutations may have developed from exposure to different TMEs.

We report that PD-L1 expression varies by metastatic site and is not concordant with TMB, but matches the organ-specific immune TME as determined by our RNAseq analysis, with better accuracy. For example, we showed LvMs have the lowest percentage of PD-L1^+^ tumors despite demonstrating high TMB. These findings demonstrate the incomplete picture of using PD-L1 status and/or TMB as a predictive marker for response to immunotherapy and the need to consider dominant site of metastasis and immune cell topography when evaluating a patient’s eligibility for checkpoint inhibition. For example, in a patient in whom disease progression is isolated to the liver, it may be prudent to ensure the tumor biopsy tested for PD-L1 is also from the liver as this may more accurately predict response to checkpoint inhibition. In our TME analysis, we demonstrated a significantly lower infiltration of adaptive immune cells notably CD4^+^ and CD8^+^ T-cells in LvM compared to BrT and other distant metastatic sites, which is consistent with findings from previous studies looking at liver metastasis [20, 32]. We also saw approximately six times the percentage of inflamed T-cells in BrT and NLvM compared to LvM and approximately a 15-fold increase in the percentage of inflamed T-cells in nodal metastasis compared to LvM. (Table 8; Supplemental Table 8) This finding is possibly explained by the role of liver sinusoidal endothelial cells (LSECs) in liver metastasis. LSECs interact with naïve CD8^+^ T-cells and subsequently suppress their cytokine production and induces T-cell immune tolerance [33]. These hepatic nonparenchymal cells also “veto” the conventional DCs antigen presentation by reducing IL-12 secretion and stunt CD8^+^ T-cell priming [34, 35]. Furthermore, our gene expression findings show elevated levels of TGF-beta1 in LvM (LvM: median value 24.86 vs. 23.31 TPM, *q*-value 0.004) and decreased levels of TLR1, TLR2, and TLR8 in LvM compared to BrT. This may be explained by the role of immune regulatory cytokines such as tumor Growth Factor-*b* (TGF-*b*), which is an important cytokine produced by Kupfer cells, hepatic stellate cells, and LSECs which induces CD4^+^CD25^+^FOXP3^+^ T-reg and epithelial to mesenchymal transition (EMT) important in the metastatic process, and negative regulators of toll-like receptor signaling, which lead to a hyporesponsive immune state in the liver [13, 36]. It may also be attributed to inactivation of effector T-cell and incomplete activation of CD8^+^ T-cells [37]. In addition, within the liver, there appears to be antigen-specific apoptotic pathways involving the Fas ligand and CD11b that could lead to CD8^+^ T-cell apoptosis [38]. Evaluation of immune modulators that target these cytokines may be worthwhile when considering combination therapies with checkpoint inhibitors in patients with liver metastases. Furthermore, additional analysis evaluating the gene expression of CD4^+^ and CD8^+^ T-cells could help identify other genes contributing to CD4^+^ and CD8^+^ T-cell depletion in LvM. Our analysis revealed significantly lower T-regs in LvMs compared to BrTs and on investigation of signature genes in T-regs, decreased expression of nearly all genes including CTLA-4 and FoxP3, similar to findings from the AURORA study which showed decreased T-regs in metastatic sites and another study on matched pair comparisons of TNBC patients (GSE110590) that showed abundance of T-regs was decreased in the liver and other metastatic sites [20, 39]. However, the rationale behind the decrease abundance of T-regs in LvM is poorly understood, as in other primary cancers such as hepatocellular carcinoma, metastatic colorectal and prostate cancer to the liver, there have been reported to be higher numbers of T-regs in the liver and associated genes such as LAG3, GITR, ICOS, and CTLA-4, which have correlated with worse outcomes [40–42]. One thought could be how T-regs behave in the LvM compared to most other sites of tumors. T-reg differentiation is a result of thymus-derived Tregs (tTregs) compromising the majority of intratumoral Tregs, and induced Tregs (iTregs) that develop in peripheral tissues from conventional T-cells (Tconv) via a TGF-beta-dependent manner [43]. LvM suppress systemic anti-tumor immunity which likely reduces tTreg differentiation from CD4-single positive (CD4-SP+) thymocytes reducing T-regs in peripheral blood circulation [43]. LvM reduce Tconv prevalence and overwhelm the liver’s natural physiological cytokine production leading towards differentiation towards iTregs, which have been shown to produce the immunosuppressive cytokine IL-10 and increase FoxP3 following stimulation [43]. Of note, in our analysis we show increased TGF-beta expression in LvM compared to BrT. Furthermore, T-regs also may interact with CD11b^+^ monocytes to alter distant tumor immunity and subsequently suppress PD-1 and CTLA-4 co-expression in CD8^+^ T-cells in LvM [44]. These differences may contribute to the lack of response to immune checkpoint inhibition observed in patients with LvMs [20, 45]. We also reported differences in TAMs which are known to affect tumor growth, angiogenesis, immune regulation, metastasis, and chemoresistance [46]. M1-like macrophages secrete proinflammatory anticancer cytokines while M2-like macrophages favor pro-tumor functions leading to angiogenesis, immune-suppression, and tissue repair [46]. Of particular interest is the increase in M2-like TAMs in LvMs and NLvMs, and increased M2-polarizing CCL2 gene expression in LvMs which supports the immunosuppressive TME that has been described within the liver. Taken together, these data suggest that the TME in LvM maybe driven by innate immune cells that undergo phenotypic changes and/or molecular switching and less so by adaptive immune cells including T-regs. Furthermore, compared to previous data, our study shows differences in immune cell abundance by type of macrophage; we saw greater M1 macrophages in LvM and NLvM relative to BrT while we saw decreased M2 macrophages in NLvM relative to BrT [19, 20]. This highlights the likely differences in role of different phenotypes of TAMs in the metastatic TME. In contrast to the immune depleted microenvironment of LvM, it is important to note the enriched immune TME in AxMs. Our results demonstrate that the AxMs associated with increased infiltration with CD4^+^, CD8^+^, T-regs and B-cells compared with BrT biopsies. We postulate that increased immune response seen in tumor draining lymph nodes (TDLN) represents an acute inflammatory reaction to mitigate distant spread. Previous research comparing anti-tumor microenvironment in TDLN vs. distant LN showed increased CD8^+^ T-cells revealed a more robust anti-tumor immune response in TDLN [47–49]. Similarly, Rye et al. showed that metastatic LNs had higher frequency of activated T-regs and dysfunctional T-cell immunoreceptor with Ig and ITIM domains (TIGIT)-positive T-cells with suppressed TCR signaling suggesting effector T-cells exhaustion as compared to non-metastatic sentinel LNs [50]. The study elucidated that tumor foci within a lymph node had higher CD8^+^ T-cells infiltration compared with extratumoral region. The variations of T-cell residence within lymph nodes correlate with tumor infiltration suggesting that tumor cells drive T effector cells toward exhaustion and promote immunosuppression by recruitment or increase in T-regs [50]. We hypothesize that increased adaptive immune cells in early metastases including active involvement of T-regs, such as the sentinel followed by non-sentinel axillary lymph nodes, depletes the immune response leading to a more barren immune TME in distant metastasis particularly in the liver. Of note, the clinical survival significance of AxM involvement remains unknown; there was no significant survival difference in patients who received node dissection in the NSABP-04 trial and in the SOUND trial in patients who had a sentinel node excision. [51, 52] However, there are still certain patients who benefit from node dissection such as young patients in which chemotherapy is associated with survival benefit in node positive disease and in ER+ breast cancer in which CDK4/6 inhibitor may be of benefit [53, 54]. There also have been significant advances in the use of ICI with the approval of pembrolizumab both in the neoadjuvant and metastatic setting in TNBC and the approval of antibody drug conjugates such as trastuzumab deruxtecan and sacituzumab govitecan [55, 56]. Thus, the contrast in TME between the AxM and LvM warrant further investigation into consideration of current ICI use based on site of metastasis and drug development of immune modulating agents that target the innate immune system to boost response in patients with LvM. Study limitations include the lack of available information about pathological stage along with treatments received and response to treatment. It is worth noting that next generation sequencing is typically used in patients with metastatic disease and thus we presume most patients in this study have metastatic disease. Outcome data are available for newer data sets through Caris however these studies were performed prior to this availability. Additionally, the samples were unpaired, and site-to-site comparisons in same patient could not be performed. Moreover, our cellular characterization of the immune TME relied on gene expression only, and thus future validation at the protein/cellular level can provide further confirmation of cellular identity. Our findings highlight the discordance of our current biomarkers—PD-L1 and TMB especially in LvM and AxM and differences in components of the immune TME between metastatic sites that merit investigation towards strategies that consider site of metastatic disease and measurements of specific immune cell infiltration.

### Supplementary Information

Below is the link to the electronic supplementary material.

Supplementary file1 (PPTX 563 KB)

## Data Availability

The aggregate summarized Caris datasets generated during and/or analyzed during the current study can be requested from corresponding authors on reasonable request. The deidentified sequencing data are owned by Caris Life Sciences. Qualified researchers can apply for access to summarized data by contacting Joanne Xiu, PhD and signing a data usage agreement.
