# Supplementary material for: Evaluation of markers of immunity in different metastatic immune microenvironments suggests more suppression within breast to liver metastases in breast cancer
Source: Breast Cancer Res Treat. 2024 Apr 20;206(2):245–59. doi: 10.1007/s10549-024-07295-w (PMC11182800; doi:10.1007/s10549-024-07295-w)
Supplement: Supplementary file 1 — Supplementary file1 (PPTX 563 KB) [file 10549_2024_7295_MOESM1_ESM.pptx]

## Slide 1
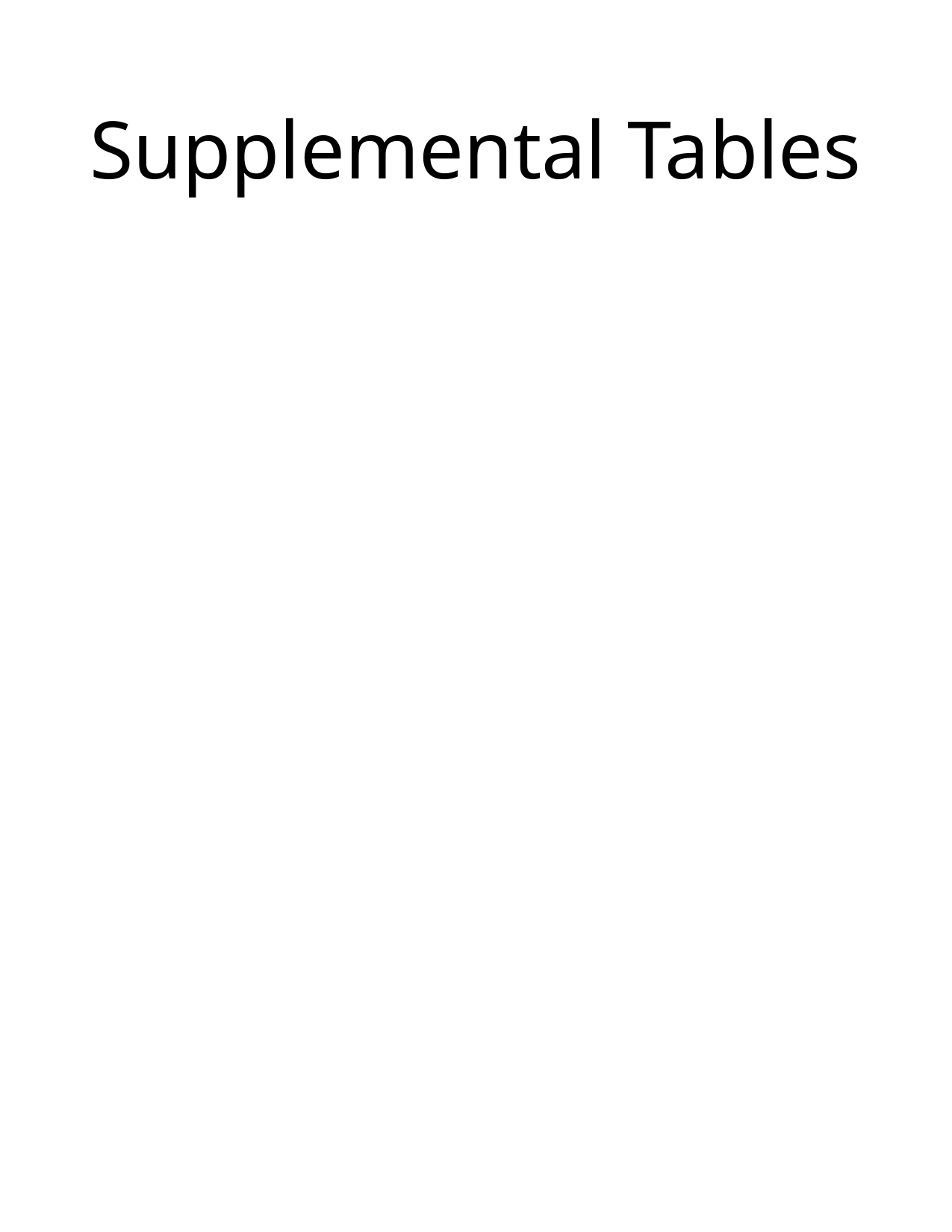

# Supplemental Tables

## Slide 2
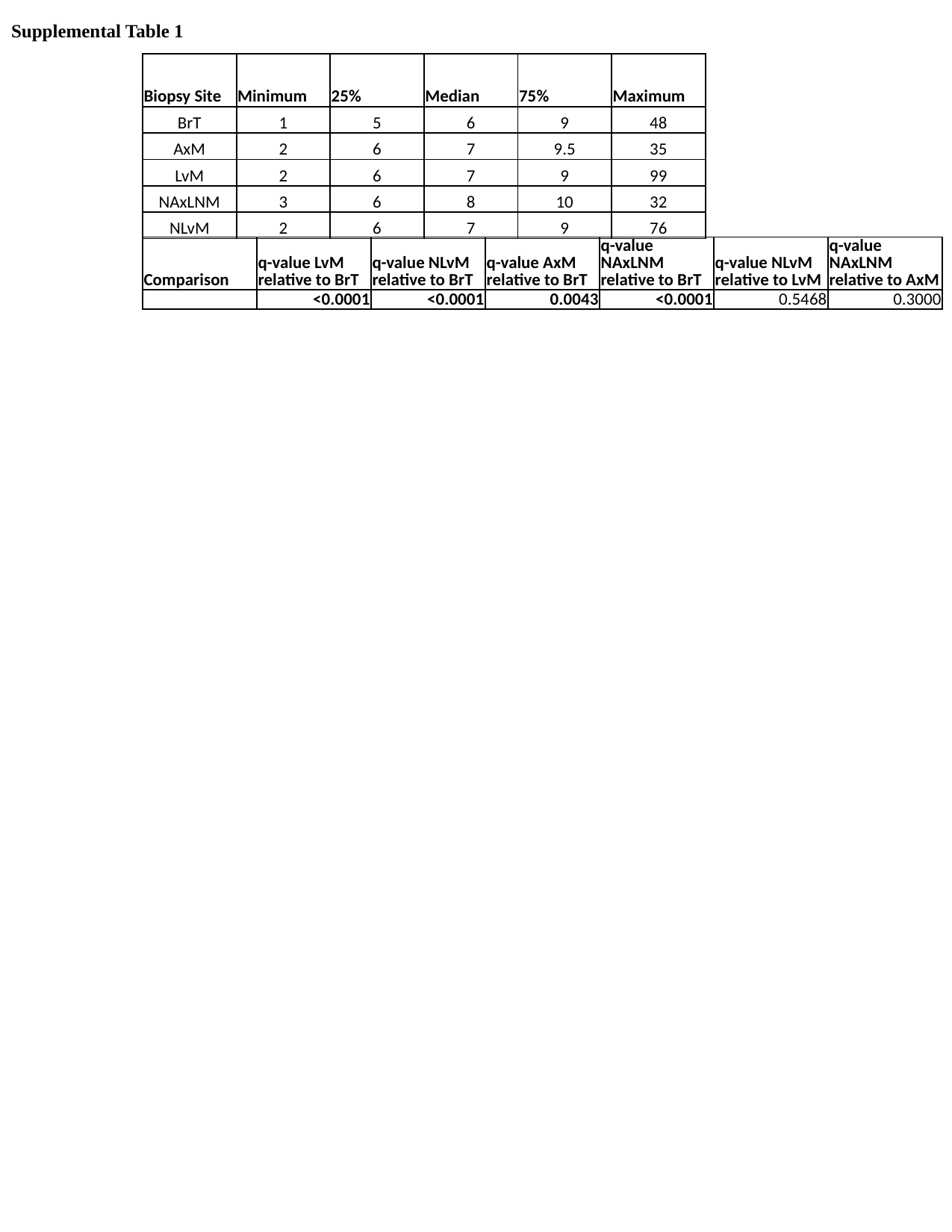

Supplemental Table 1
| Biopsy Site | Minimum | 25% | Median | 75% | Maximum |
| --- | --- | --- | --- | --- | --- |
| BrT | 1 | 5 | 6 | 9 | 48 |
| AxM | 2 | 6 | 7 | 9.5 | 35 |
| LvM | 2 | 6 | 7 | 9 | 99 |
| NAxLNM | 3 | 6 | 8 | 10 | 32 |
| NLvM | 2 | 6 | 7 | 9 | 76 |
| Comparison | q-value LvM relative to BrT | q-value NLvM relative to BrT | q-value AxM relative to BrT | q-value NAxLNM relative to BrT | q-value NLvM relative to LvM | q-value NAxLNM relative to AxM |
| --- | --- | --- | --- | --- | --- | --- |
| | <0.0001 | <0.0001 | 0.0043 | <0.0001 | 0.5468 | 0.3000 |

## Slide 3
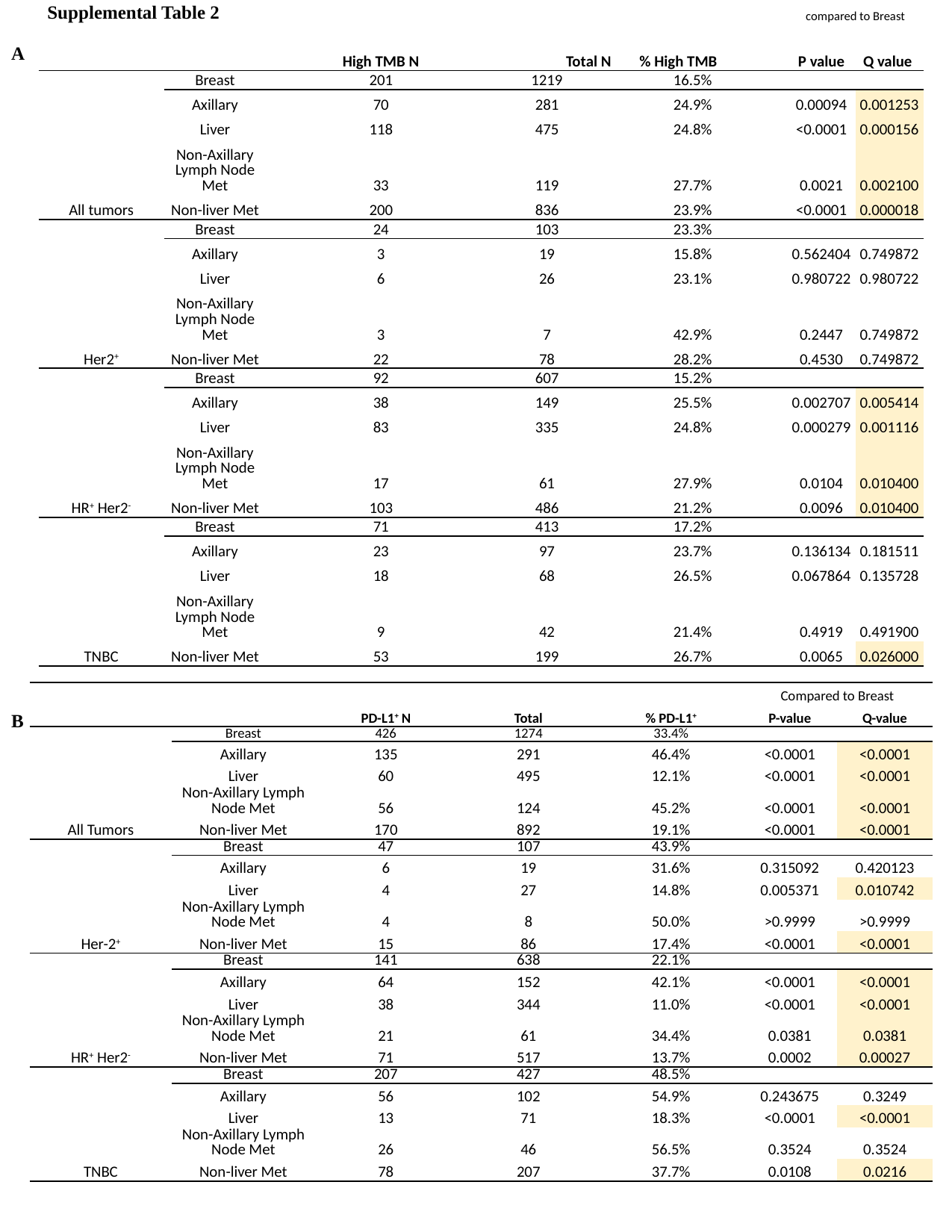

| | | | | | compared to Breast | |
| --- | --- | --- | --- | --- | --- | --- |
| | | High TMB N | Total N % High TMB | | P value | Q value |
| All tumors | Breast | 201 | 1219 | 16.5% | | |
| | Axillary | 70 | 281 | 24.9% | 0.00094 | 0.001253 |
| | Liver | 118 | 475 | 24.8% | <0.0001 | 0.000156 |
| | Non-Axillary Lymph Node Met | 33 | 119 | 27.7% | 0.0021 | 0.002100 |
| | Non-liver Met | 200 | 836 | 23.9% | <0.0001 | 0.000018 |
| Her2+ | Breast | 24 | 103 | 23.3% | | |
| | Axillary | 3 | 19 | 15.8% | 0.562404 | 0.749872 |
| | Liver | 6 | 26 | 23.1% | 0.980722 | 0.980722 |
| | Non-Axillary Lymph Node Met | 3 | 7 | 42.9% | 0.2447 | 0.749872 |
| | Non-liver Met | 22 | 78 | 28.2% | 0.4530 | 0.749872 |
| HR+ Her2- | Breast | 92 | 607 | 15.2% | | |
| | Axillary | 38 | 149 | 25.5% | 0.002707 | 0.005414 |
| | Liver | 83 | 335 | 24.8% | 0.000279 | 0.001116 |
| | Non-Axillary Lymph Node Met | 17 | 61 | 27.9% | 0.0104 | 0.010400 |
| | Non-liver Met | 103 | 486 | 21.2% | 0.0096 | 0.010400 |
| TNBC | Breast | 71 | 413 | 17.2% | | |
| | Axillary | 23 | 97 | 23.7% | 0.136134 | 0.181511 |
| | Liver | 18 | 68 | 26.5% | 0.067864 | 0.135728 |
| | Non-Axillary Lymph Node Met | 9 | 42 | 21.4% | 0.4919 | 0.491900 |
| | Non-liver Met | 53 | 199 | 26.7% | 0.0065 | 0.026000 |
Supplemental Table 2
# A
| | | | | | Compared to Breast | |
| --- | --- | --- | --- | --- | --- | --- |
| | | PD-L1+ N | Total | % PD-L1+ | P-value | Q-value |
| All Tumors | Breast | 426 | 1274 | 33.4% | | |
| | Axillary | 135 | 291 | 46.4% | <0.0001 | <0.0001 |
| | Liver | 60 | 495 | 12.1% | <0.0001 | <0.0001 |
| | Non-Axillary Lymph Node Met | 56 | 124 | 45.2% | <0.0001 | <0.0001 |
| | Non-liver Met | 170 | 892 | 19.1% | <0.0001 | <0.0001 |
| Her-2+ | Breast | 47 | 107 | 43.9% | | |
| | Axillary | 6 | 19 | 31.6% | 0.315092 | 0.420123 |
| | Liver | 4 | 27 | 14.8% | 0.005371 | 0.010742 |
| | Non-Axillary Lymph Node Met | 4 | 8 | 50.0% | >0.9999 | >0.9999 |
| | Non-liver Met | 15 | 86 | 17.4% | <0.0001 | <0.0001 |
| HR+ Her2- | Breast | 141 | 638 | 22.1% | | |
| | Axillary | 64 | 152 | 42.1% | <0.0001 | <0.0001 |
| | Liver | 38 | 344 | 11.0% | <0.0001 | <0.0001 |
| | Non-Axillary Lymph Node Met | 21 | 61 | 34.4% | 0.0381 | 0.0381 |
| | Non-liver Met | 71 | 517 | 13.7% | 0.0002 | 0.00027 |
| TNBC | Breast | 207 | 427 | 48.5% | | |
| | Axillary | 56 | 102 | 54.9% | 0.243675 | 0.3249 |
| | Liver | 13 | 71 | 18.3% | <0.0001 | <0.0001 |
| | Non-Axillary Lymph Node Met | 26 | 46 | 56.5% | 0.3524 | 0.3524 |
| | Non-liver Met | 78 | 207 | 37.7% | 0.0108 | 0.0216 |
B

## Slide 4
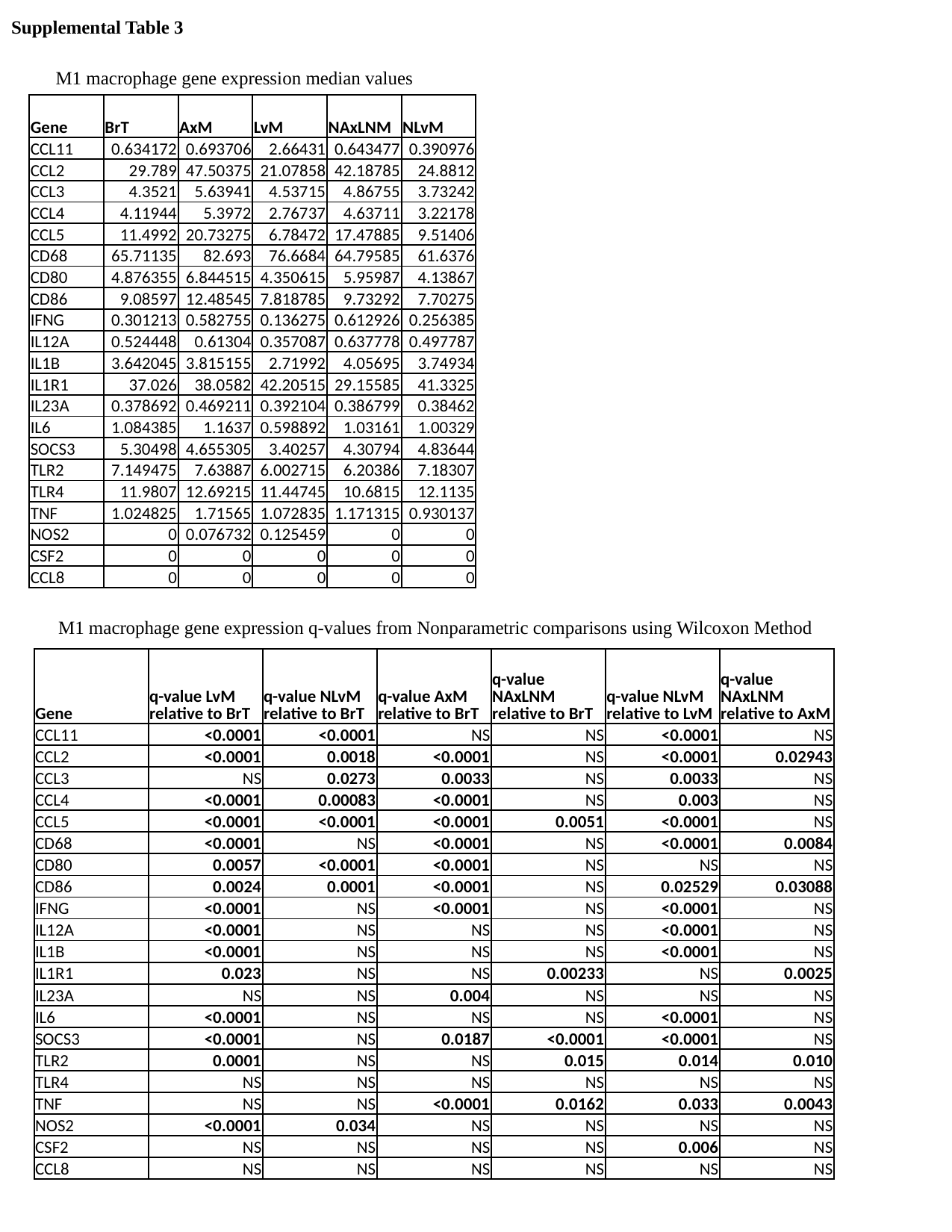

Supplemental Table 3
M1 macrophage gene expression median values
| Gene | BrT | AxM | LvM | NAxLNM | NLvM |
| --- | --- | --- | --- | --- | --- |
| CCL11 | 0.634172 | 0.693706 | 2.66431 | 0.643477 | 0.390976 |
| CCL2 | 29.789 | 47.50375 | 21.07858 | 42.18785 | 24.8812 |
| CCL3 | 4.3521 | 5.63941 | 4.53715 | 4.86755 | 3.73242 |
| CCL4 | 4.11944 | 5.3972 | 2.76737 | 4.63711 | 3.22178 |
| CCL5 | 11.4992 | 20.73275 | 6.78472 | 17.47885 | 9.51406 |
| CD68 | 65.71135 | 82.693 | 76.6684 | 64.79585 | 61.6376 |
| CD80 | 4.876355 | 6.844515 | 4.350615 | 5.95987 | 4.13867 |
| CD86 | 9.08597 | 12.48545 | 7.818785 | 9.73292 | 7.70275 |
| IFNG | 0.301213 | 0.582755 | 0.136275 | 0.612926 | 0.256385 |
| IL12A | 0.524448 | 0.61304 | 0.357087 | 0.637778 | 0.497787 |
| IL1B | 3.642045 | 3.815155 | 2.71992 | 4.05695 | 3.74934 |
| IL1R1 | 37.026 | 38.0582 | 42.20515 | 29.15585 | 41.3325 |
| IL23A | 0.378692 | 0.469211 | 0.392104 | 0.386799 | 0.38462 |
| IL6 | 1.084385 | 1.1637 | 0.598892 | 1.03161 | 1.00329 |
| SOCS3 | 5.30498 | 4.655305 | 3.40257 | 4.30794 | 4.83644 |
| TLR2 | 7.149475 | 7.63887 | 6.002715 | 6.20386 | 7.18307 |
| TLR4 | 11.9807 | 12.69215 | 11.44745 | 10.6815 | 12.1135 |
| TNF | 1.024825 | 1.71565 | 1.072835 | 1.171315 | 0.930137 |
| NOS2 | 0 | 0.076732 | 0.125459 | 0 | 0 |
| CSF2 | 0 | 0 | 0 | 0 | 0 |
| CCL8 | 0 | 0 | 0 | 0 | 0 |
M1 macrophage gene expression q-values from Nonparametric comparisons using Wilcoxon Method
| Gene | q-value LvM relative to BrT | q-value NLvM relative to BrT | q-value AxM relative to BrT | q-value NAxLNM relative to BrT | q-value NLvM relative to LvM | q-value NAxLNM relative to AxM |
| --- | --- | --- | --- | --- | --- | --- |
| CCL11 | <0.0001 | <0.0001 | NS | NS | <0.0001 | NS |
| CCL2 | <0.0001 | 0.0018 | <0.0001 | NS | <0.0001 | 0.02943 |
| CCL3 | NS | 0.0273 | 0.0033 | NS | 0.0033 | NS |
| CCL4 | <0.0001 | 0.00083 | <0.0001 | NS | 0.003 | NS |
| CCL5 | <0.0001 | <0.0001 | <0.0001 | 0.0051 | <0.0001 | NS |
| CD68 | <0.0001 | NS | <0.0001 | NS | <0.0001 | 0.0084 |
| CD80 | 0.0057 | <0.0001 | <0.0001 | NS | NS | NS |
| CD86 | 0.0024 | 0.0001 | <0.0001 | NS | 0.02529 | 0.03088 |
| IFNG | <0.0001 | NS | <0.0001 | NS | <0.0001 | NS |
| IL12A | <0.0001 | NS | NS | NS | <0.0001 | NS |
| IL1B | <0.0001 | NS | NS | NS | <0.0001 | NS |
| IL1R1 | 0.023 | NS | NS | 0.00233 | NS | 0.0025 |
| IL23A | NS | NS | 0.004 | NS | NS | NS |
| IL6 | <0.0001 | NS | NS | NS | <0.0001 | NS |
| SOCS3 | <0.0001 | NS | 0.0187 | <0.0001 | <0.0001 | NS |
| TLR2 | 0.0001 | NS | NS | 0.015 | 0.014 | 0.010 |
| TLR4 | NS | NS | NS | NS | NS | NS |
| TNF | NS | NS | <0.0001 | 0.0162 | 0.033 | 0.0043 |
| NOS2 | <0.0001 | 0.034 | NS | NS | NS | NS |
| CSF2 | NS | NS | NS | NS | 0.006 | NS |
| CCL8 | NS | NS | NS | NS | NS | NS |

## Slide 5
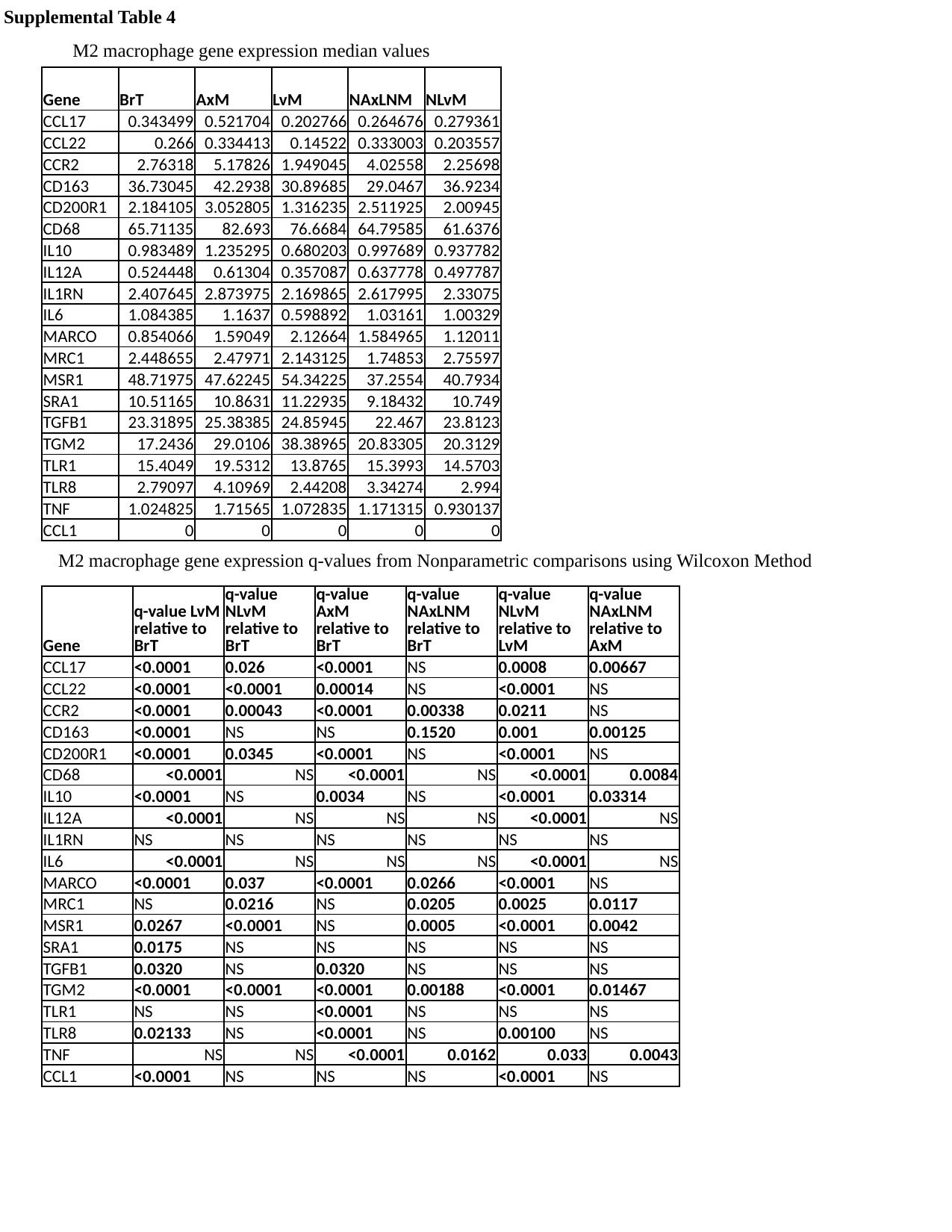

Supplemental Table 4
M2 macrophage gene expression median values
| Gene | BrT | AxM | LvM | NAxLNM | NLvM |
| --- | --- | --- | --- | --- | --- |
| CCL17 | 0.343499 | 0.521704 | 0.202766 | 0.264676 | 0.279361 |
| CCL22 | 0.266 | 0.334413 | 0.14522 | 0.333003 | 0.203557 |
| CCR2 | 2.76318 | 5.17826 | 1.949045 | 4.02558 | 2.25698 |
| CD163 | 36.73045 | 42.2938 | 30.89685 | 29.0467 | 36.9234 |
| CD200R1 | 2.184105 | 3.052805 | 1.316235 | 2.511925 | 2.00945 |
| CD68 | 65.71135 | 82.693 | 76.6684 | 64.79585 | 61.6376 |
| IL10 | 0.983489 | 1.235295 | 0.680203 | 0.997689 | 0.937782 |
| IL12A | 0.524448 | 0.61304 | 0.357087 | 0.637778 | 0.497787 |
| IL1RN | 2.407645 | 2.873975 | 2.169865 | 2.617995 | 2.33075 |
| IL6 | 1.084385 | 1.1637 | 0.598892 | 1.03161 | 1.00329 |
| MARCO | 0.854066 | 1.59049 | 2.12664 | 1.584965 | 1.12011 |
| MRC1 | 2.448655 | 2.47971 | 2.143125 | 1.74853 | 2.75597 |
| MSR1 | 48.71975 | 47.62245 | 54.34225 | 37.2554 | 40.7934 |
| SRA1 | 10.51165 | 10.8631 | 11.22935 | 9.18432 | 10.749 |
| TGFB1 | 23.31895 | 25.38385 | 24.85945 | 22.467 | 23.8123 |
| TGM2 | 17.2436 | 29.0106 | 38.38965 | 20.83305 | 20.3129 |
| TLR1 | 15.4049 | 19.5312 | 13.8765 | 15.3993 | 14.5703 |
| TLR8 | 2.79097 | 4.10969 | 2.44208 | 3.34274 | 2.994 |
| TNF | 1.024825 | 1.71565 | 1.072835 | 1.171315 | 0.930137 |
| CCL1 | 0 | 0 | 0 | 0 | 0 |
M2 macrophage gene expression q-values from Nonparametric comparisons using Wilcoxon Method
| Gene | q-value LvM relative to BrT | q-value NLvM relative to BrT | q-value AxM relative to BrT | q-value NAxLNM relative to BrT | q-value NLvM relative to LvM | q-value NAxLNM relative to AxM |
| --- | --- | --- | --- | --- | --- | --- |
| CCL17 | <0.0001 | 0.026 | <0.0001 | NS | 0.0008 | 0.00667 |
| CCL22 | <0.0001 | <0.0001 | 0.00014 | NS | <0.0001 | NS |
| CCR2 | <0.0001 | 0.00043 | <0.0001 | 0.00338 | 0.0211 | NS |
| CD163 | <0.0001 | NS | NS | 0.1520 | 0.001 | 0.00125 |
| CD200R1 | <0.0001 | 0.0345 | <0.0001 | NS | <0.0001 | NS |
| CD68 | <0.0001 | NS | <0.0001 | NS | <0.0001 | 0.0084 |
| IL10 | <0.0001 | NS | 0.0034 | NS | <0.0001 | 0.03314 |
| IL12A | <0.0001 | NS | NS | NS | <0.0001 | NS |
| IL1RN | NS | NS | NS | NS | NS | NS |
| IL6 | <0.0001 | NS | NS | NS | <0.0001 | NS |
| MARCO | <0.0001 | 0.037 | <0.0001 | 0.0266 | <0.0001 | NS |
| MRC1 | NS | 0.0216 | NS | 0.0205 | 0.0025 | 0.0117 |
| MSR1 | 0.0267 | <0.0001 | NS | 0.0005 | <0.0001 | 0.0042 |
| SRA1 | 0.0175 | NS | NS | NS | NS | NS |
| TGFB1 | 0.0320 | NS | 0.0320 | NS | NS | NS |
| TGM2 | <0.0001 | <0.0001 | <0.0001 | 0.00188 | <0.0001 | 0.01467 |
| TLR1 | NS | NS | <0.0001 | NS | NS | NS |
| TLR8 | 0.02133 | NS | <0.0001 | NS | 0.00100 | NS |
| TNF | NS | NS | <0.0001 | 0.0162 | 0.033 | 0.0043 |
| CCL1 | <0.0001 | NS | NS | NS | <0.0001 | NS |

## Slide 6
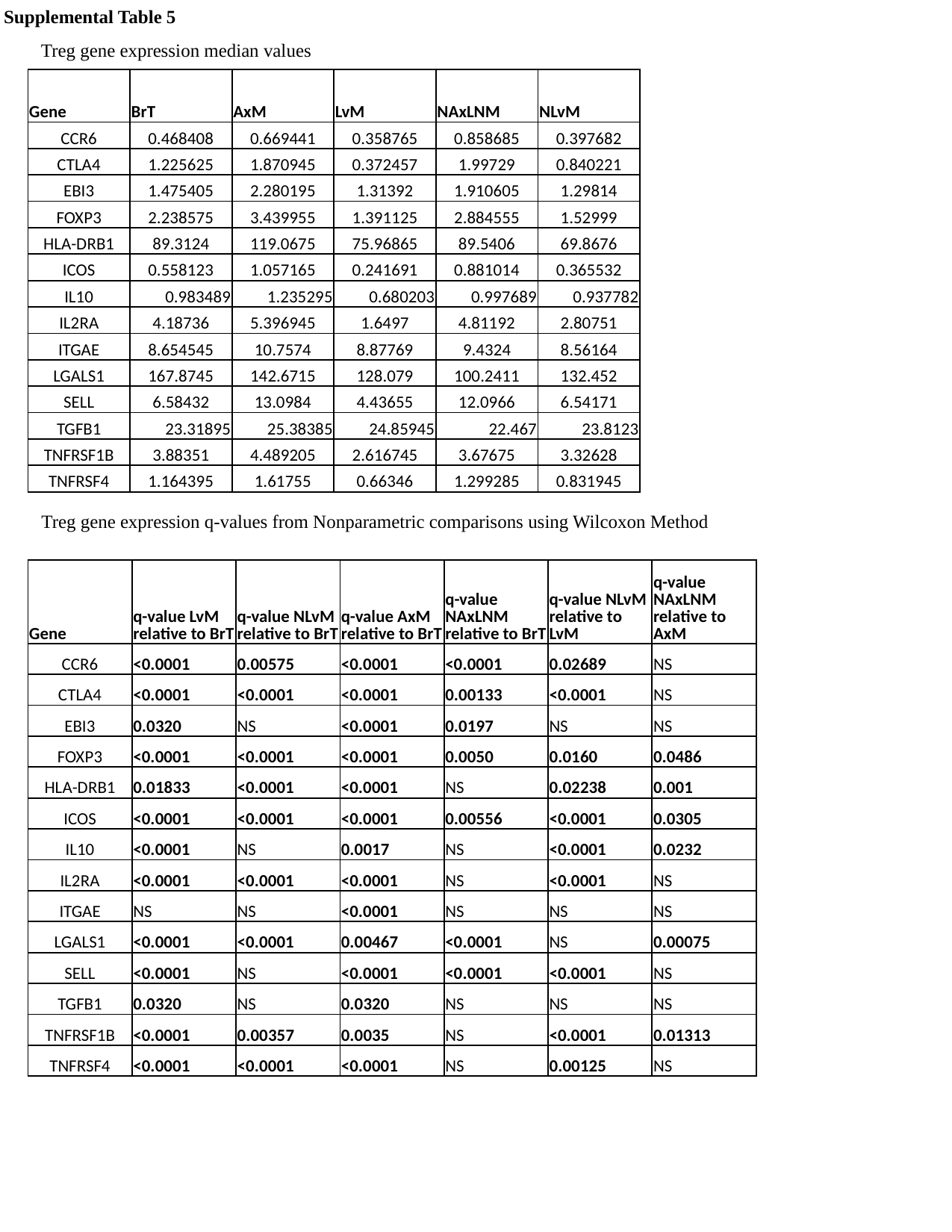

Supplemental Table 5
Treg gene expression median values
| Gene | BrT | AxM | LvM | NAxLNM | NLvM |
| --- | --- | --- | --- | --- | --- |
| CCR6 | 0.468408 | 0.669441 | 0.358765 | 0.858685 | 0.397682 |
| CTLA4 | 1.225625 | 1.870945 | 0.372457 | 1.99729 | 0.840221 |
| EBI3 | 1.475405 | 2.280195 | 1.31392 | 1.910605 | 1.29814 |
| FOXP3 | 2.238575 | 3.439955 | 1.391125 | 2.884555 | 1.52999 |
| HLA-DRB1 | 89.3124 | 119.0675 | 75.96865 | 89.5406 | 69.8676 |
| ICOS | 0.558123 | 1.057165 | 0.241691 | 0.881014 | 0.365532 |
| IL10 | 0.983489 | 1.235295 | 0.680203 | 0.997689 | 0.937782 |
| IL2RA | 4.18736 | 5.396945 | 1.6497 | 4.81192 | 2.80751 |
| ITGAE | 8.654545 | 10.7574 | 8.87769 | 9.4324 | 8.56164 |
| LGALS1 | 167.8745 | 142.6715 | 128.079 | 100.2411 | 132.452 |
| SELL | 6.58432 | 13.0984 | 4.43655 | 12.0966 | 6.54171 |
| TGFB1 | 23.31895 | 25.38385 | 24.85945 | 22.467 | 23.8123 |
| TNFRSF1B | 3.88351 | 4.489205 | 2.616745 | 3.67675 | 3.32628 |
| TNFRSF4 | 1.164395 | 1.61755 | 0.66346 | 1.299285 | 0.831945 |
Treg gene expression q-values from Nonparametric comparisons using Wilcoxon Method
| Gene | q-value LvM relative to BrT | q-value NLvM relative to BrT | q-value AxM relative to BrT | q-value NAxLNM relative to BrT | q-value NLvM relative to LvM | q-value NAxLNM relative to AxM |
| --- | --- | --- | --- | --- | --- | --- |
| CCR6 | <0.0001 | 0.00575 | <0.0001 | <0.0001 | 0.02689 | NS |
| CTLA4 | <0.0001 | <0.0001 | <0.0001 | 0.00133 | <0.0001 | NS |
| EBI3 | 0.0320 | NS | <0.0001 | 0.0197 | NS | NS |
| FOXP3 | <0.0001 | <0.0001 | <0.0001 | 0.0050 | 0.0160 | 0.0486 |
| HLA-DRB1 | 0.01833 | <0.0001 | <0.0001 | NS | 0.02238 | 0.001 |
| ICOS | <0.0001 | <0.0001 | <0.0001 | 0.00556 | <0.0001 | 0.0305 |
| IL10 | <0.0001 | NS | 0.0017 | NS | <0.0001 | 0.0232 |
| IL2RA | <0.0001 | <0.0001 | <0.0001 | NS | <0.0001 | NS |
| ITGAE | NS | NS | <0.0001 | NS | NS | NS |
| LGALS1 | <0.0001 | <0.0001 | 0.00467 | <0.0001 | NS | 0.00075 |
| SELL | <0.0001 | NS | <0.0001 | <0.0001 | <0.0001 | NS |
| TGFB1 | 0.0320 | NS | 0.0320 | NS | NS | NS |
| TNFRSF1B | <0.0001 | 0.00357 | 0.0035 | NS | <0.0001 | 0.01313 |
| TNFRSF4 | <0.0001 | <0.0001 | <0.0001 | NS | 0.00125 | NS |

## Slide 7
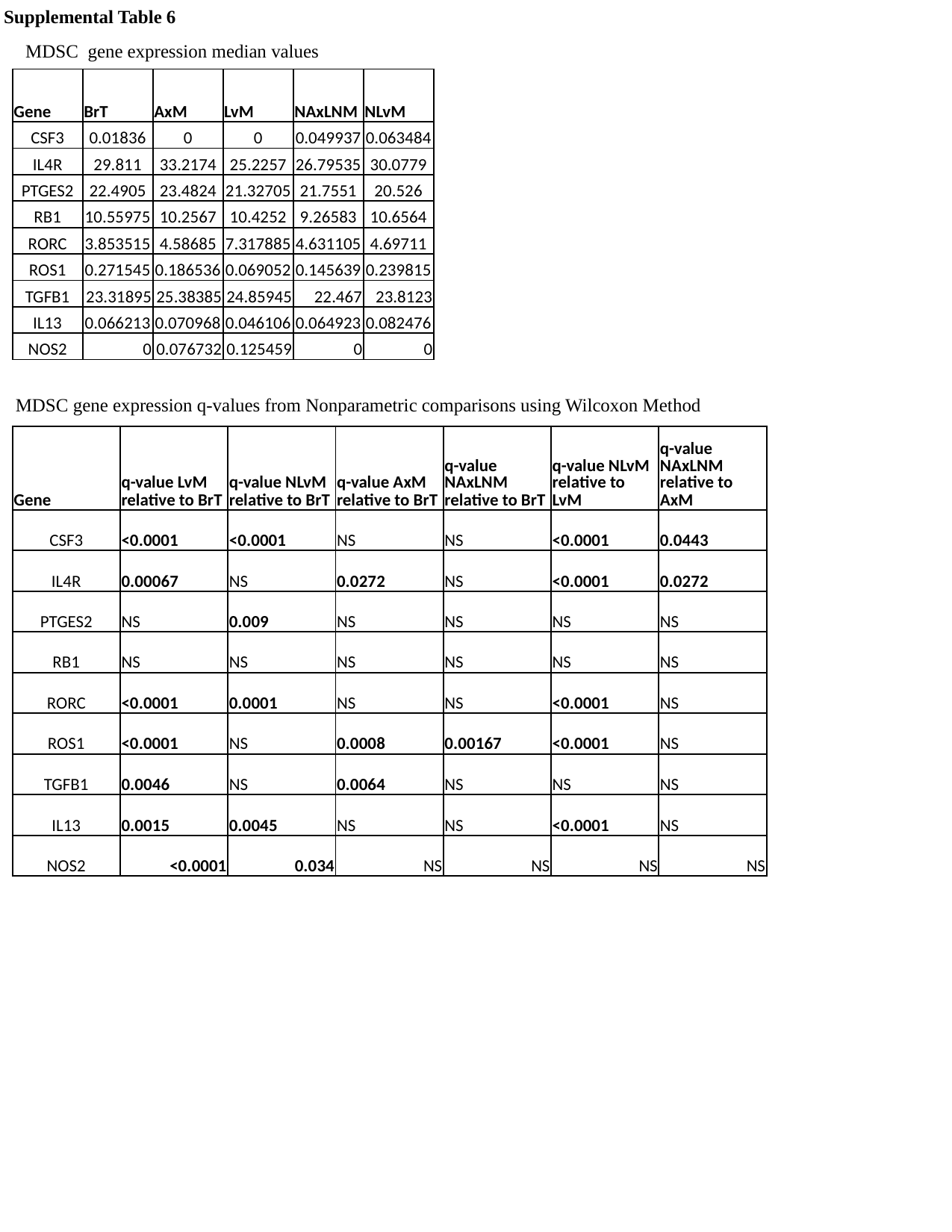

Supplemental Table 6
MDSC gene expression median values
| Gene | BrT | AxM | LvM | NAxLNM | NLvM |
| --- | --- | --- | --- | --- | --- |
| CSF3 | 0.01836 | 0 | 0 | 0.049937 | 0.063484 |
| IL4R | 29.811 | 33.2174 | 25.2257 | 26.79535 | 30.0779 |
| PTGES2 | 22.4905 | 23.4824 | 21.32705 | 21.7551 | 20.526 |
| RB1 | 10.55975 | 10.2567 | 10.4252 | 9.26583 | 10.6564 |
| RORC | 3.853515 | 4.58685 | 7.317885 | 4.631105 | 4.69711 |
| ROS1 | 0.271545 | 0.186536 | 0.069052 | 0.145639 | 0.239815 |
| TGFB1 | 23.31895 | 25.38385 | 24.85945 | 22.467 | 23.8123 |
| IL13 | 0.066213 | 0.070968 | 0.046106 | 0.064923 | 0.082476 |
| NOS2 | 0 | 0.076732 | 0.125459 | 0 | 0 |
MDSC gene expression q-values from Nonparametric comparisons using Wilcoxon Method
| Gene | q-value LvM relative to BrT | q-value NLvM relative to BrT | q-value AxM relative to BrT | q-value NAxLNM relative to BrT | q-value NLvM relative to LvM | q-value NAxLNM relative to AxM |
| --- | --- | --- | --- | --- | --- | --- |
| CSF3 | <0.0001 | <0.0001 | NS | NS | <0.0001 | 0.0443 |
| IL4R | 0.00067 | NS | 0.0272 | NS | <0.0001 | 0.0272 |
| PTGES2 | NS | 0.009 | NS | NS | NS | NS |
| RB1 | NS | NS | NS | NS | NS | NS |
| RORC | <0.0001 | 0.0001 | NS | NS | <0.0001 | NS |
| ROS1 | <0.0001 | NS | 0.0008 | 0.00167 | <0.0001 | NS |
| TGFB1 | 0.0046 | NS | 0.0064 | NS | NS | NS |
| IL13 | 0.0015 | 0.0045 | NS | NS | <0.0001 | NS |
| NOS2 | <0.0001 | 0.034 | NS | NS | NS | NS |

## Slide 8
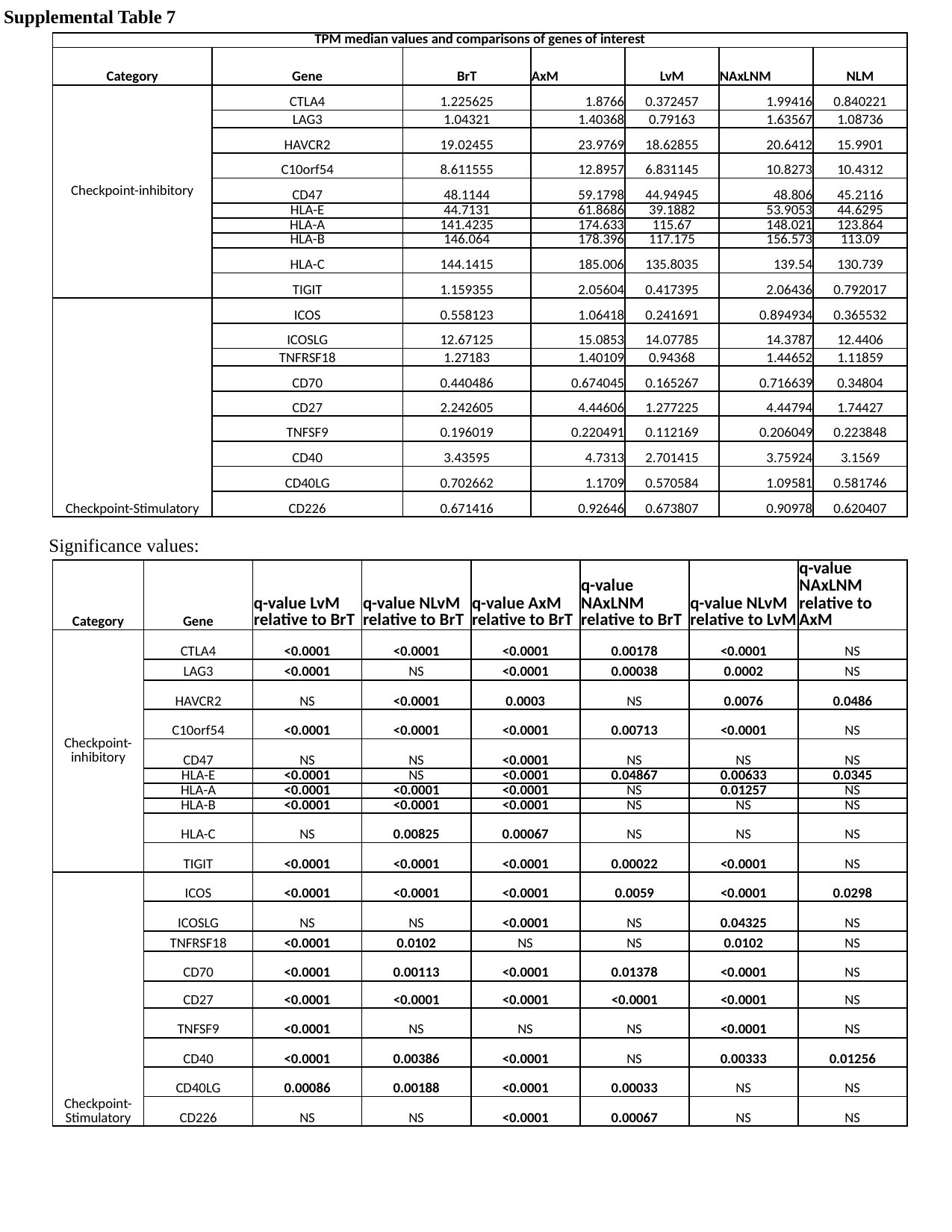

Supplemental Table 7
| TPM median values and comparisons of genes of interest | | | | | | |
| --- | --- | --- | --- | --- | --- | --- |
| Category | Gene | BrT | AxM | LvM | NAxLNM | NLM |
| Checkpoint-inhibitory | CTLA4 | 1.225625 | 1.8766 | 0.372457 | 1.99416 | 0.840221 |
| | LAG3 | 1.04321 | 1.40368 | 0.79163 | 1.63567 | 1.08736 |
| | HAVCR2 | 19.02455 | 23.9769 | 18.62855 | 20.6412 | 15.9901 |
| | C10orf54 | 8.611555 | 12.8957 | 6.831145 | 10.8273 | 10.4312 |
| | CD47 | 48.1144 | 59.1798 | 44.94945 | 48.806 | 45.2116 |
| | HLA-E | 44.7131 | 61.8686 | 39.1882 | 53.9053 | 44.6295 |
| | HLA-A | 141.4235 | 174.633 | 115.67 | 148.021 | 123.864 |
| | HLA-B | 146.064 | 178.396 | 117.175 | 156.573 | 113.09 |
| | HLA-C | 144.1415 | 185.006 | 135.8035 | 139.54 | 130.739 |
| | TIGIT | 1.159355 | 2.05604 | 0.417395 | 2.06436 | 0.792017 |
| Checkpoint-Stimulatory | ICOS | 0.558123 | 1.06418 | 0.241691 | 0.894934 | 0.365532 |
| | ICOSLG | 12.67125 | 15.0853 | 14.07785 | 14.3787 | 12.4406 |
| | TNFRSF18 | 1.27183 | 1.40109 | 0.94368 | 1.44652 | 1.11859 |
| | CD70 | 0.440486 | 0.674045 | 0.165267 | 0.716639 | 0.34804 |
| | CD27 | 2.242605 | 4.44606 | 1.277225 | 4.44794 | 1.74427 |
| | TNFSF9 | 0.196019 | 0.220491 | 0.112169 | 0.206049 | 0.223848 |
| | CD40 | 3.43595 | 4.7313 | 2.701415 | 3.75924 | 3.1569 |
| | CD40LG | 0.702662 | 1.1709 | 0.570584 | 1.09581 | 0.581746 |
| | CD226 | 0.671416 | 0.92646 | 0.673807 | 0.90978 | 0.620407 |
Significance values:
| Category | Gene | q-value LvM relative to BrT | q-value NLvM relative to BrT | q-value AxM relative to BrT | q-value NAxLNM relative to BrT | q-value NLvM relative to LvM | q-value NAxLNM relative to AxM |
| --- | --- | --- | --- | --- | --- | --- | --- |
| Checkpoint-inhibitory | CTLA4 | <0.0001 | <0.0001 | <0.0001 | 0.00178 | <0.0001 | NS |
| | LAG3 | <0.0001 | NS | <0.0001 | 0.00038 | 0.0002 | NS |
| | HAVCR2 | NS | <0.0001 | 0.0003 | NS | 0.0076 | 0.0486 |
| | C10orf54 | <0.0001 | <0.0001 | <0.0001 | 0.00713 | <0.0001 | NS |
| | CD47 | NS | NS | <0.0001 | NS | NS | NS |
| | HLA-E | <0.0001 | NS | <0.0001 | 0.04867 | 0.00633 | 0.0345 |
| | HLA-A | <0.0001 | <0.0001 | <0.0001 | NS | 0.01257 | NS |
| | HLA-B | <0.0001 | <0.0001 | <0.0001 | NS | NS | NS |
| | HLA-C | NS | 0.00825 | 0.00067 | NS | NS | NS |
| | TIGIT | <0.0001 | <0.0001 | <0.0001 | 0.00022 | <0.0001 | NS |
| Checkpoint-Stimulatory | ICOS | <0.0001 | <0.0001 | <0.0001 | 0.0059 | <0.0001 | 0.0298 |
| | ICOSLG | NS | NS | <0.0001 | NS | 0.04325 | NS |
| | TNFRSF18 | <0.0001 | 0.0102 | NS | NS | 0.0102 | NS |
| | CD70 | <0.0001 | 0.00113 | <0.0001 | 0.01378 | <0.0001 | NS |
| | CD27 | <0.0001 | <0.0001 | <0.0001 | <0.0001 | <0.0001 | NS |
| | TNFSF9 | <0.0001 | NS | NS | NS | <0.0001 | NS |
| | CD40 | <0.0001 | 0.00386 | <0.0001 | NS | 0.00333 | 0.01256 |
| | CD40LG | 0.00086 | 0.00188 | <0.0001 | 0.00033 | NS | NS |
| | CD226 | NS | NS | <0.0001 | 0.00067 | NS | NS |

## Slide 9
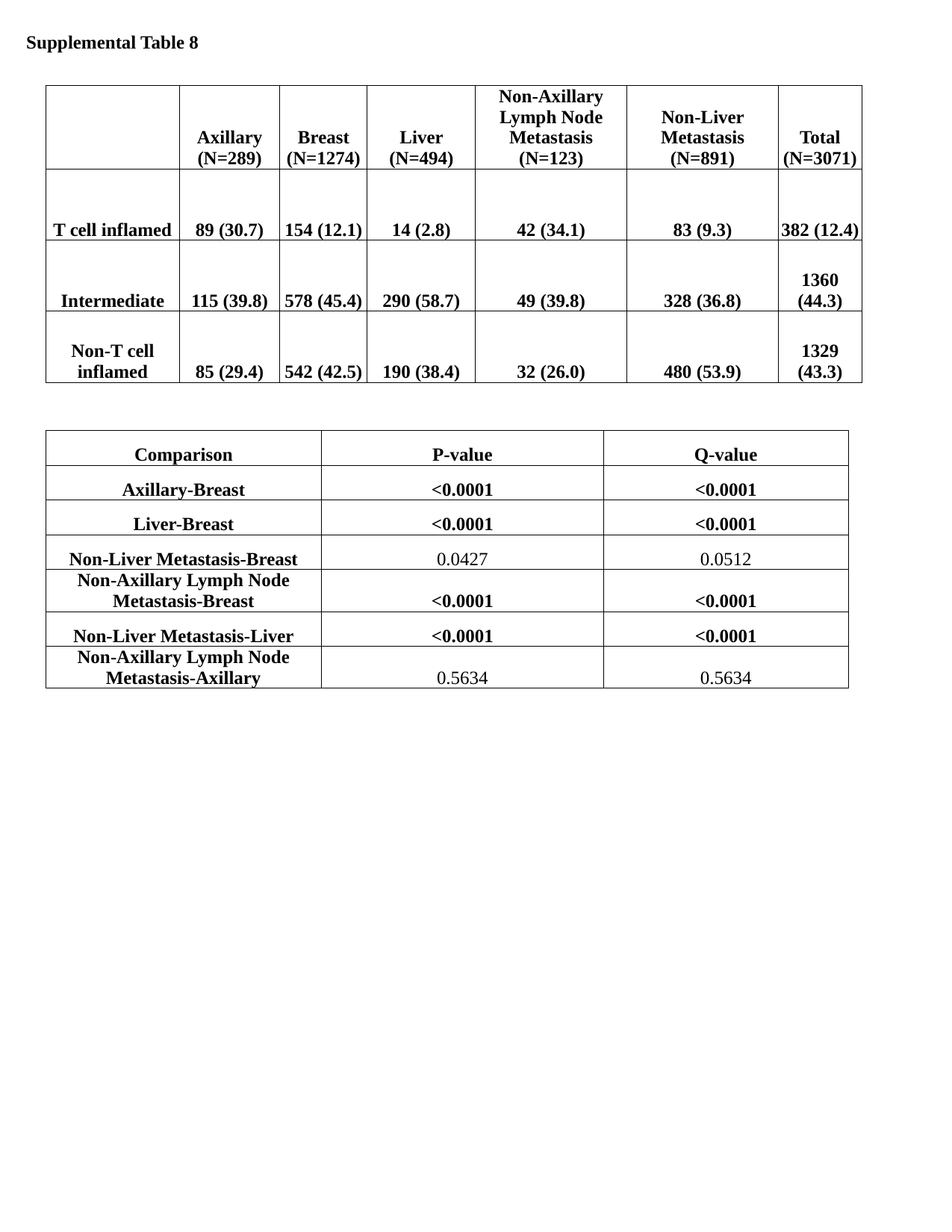

Supplemental Table 8
| | Axillary (N=289) | Breast (N=1274) | Liver (N=494) | Non-Axillary Lymph Node Metastasis (N=123) | Non-Liver Metastasis (N=891) | Total (N=3071) |
| --- | --- | --- | --- | --- | --- | --- |
| T cell inflamed | 89 (30.7) | 154 (12.1) | 14 (2.8) | 42 (34.1) | 83 (9.3) | 382 (12.4) |
| Intermediate | 115 (39.8) | 578 (45.4) | 290 (58.7) | 49 (39.8) | 328 (36.8) | 1360 (44.3) |
| Non-T cell inflamed | 85 (29.4) | 542 (42.5) | 190 (38.4) | 32 (26.0) | 480 (53.9) | 1329 (43.3) |
| Comparison | P-value | Q-value |
| --- | --- | --- |
| Axillary-Breast | <0.0001 | <0.0001 |
| Liver-Breast | <0.0001 | <0.0001 |
| Non-Liver Metastasis-Breast | 0.0427 | 0.0512 |
| Non-Axillary Lymph Node Metastasis-Breast | <0.0001 | <0.0001 |
| Non-Liver Metastasis-Liver | <0.0001 | <0.0001 |
| Non-Axillary Lymph Node Metastasis-Axillary | 0.5634 | 0.5634 |

## Slide 10
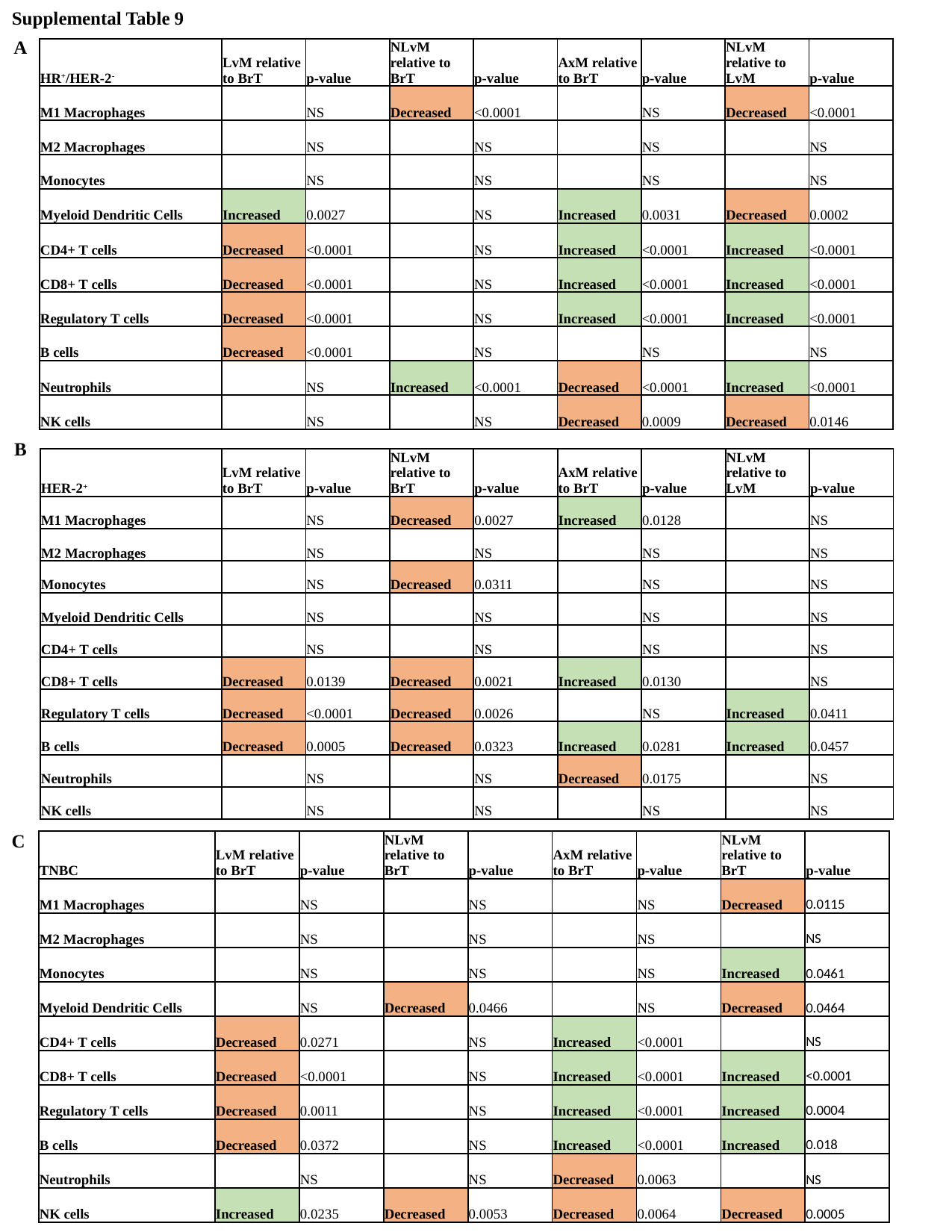

Supplemental Table 9
A
| HR+/HER-2- | LvM relative to BrT | p-value | NLvM relative to BrT | p-value | AxM relative to BrT | p-value | NLvM relative to LvM | p-value |
| --- | --- | --- | --- | --- | --- | --- | --- | --- |
| M1 Macrophages | | NS | Decreased | <0.0001 | | NS | Decreased | <0.0001 |
| M2 Macrophages | | NS | | NS | | NS | | NS |
| Monocytes | | NS | | NS | | NS | | NS |
| Myeloid Dendritic Cells | Increased | 0.0027 | | NS | Increased | 0.0031 | Decreased | 0.0002 |
| CD4+ T cells | Decreased | <0.0001 | | NS | Increased | <0.0001 | Increased | <0.0001 |
| CD8+ T cells | Decreased | <0.0001 | | NS | Increased | <0.0001 | Increased | <0.0001 |
| Regulatory T cells | Decreased | <0.0001 | | NS | Increased | <0.0001 | Increased | <0.0001 |
| B cells | Decreased | <0.0001 | | NS | | NS | | NS |
| Neutrophils | | NS | Increased | <0.0001 | Decreased | <0.0001 | Increased | <0.0001 |
| NK cells | | NS | | NS | Decreased | 0.0009 | Decreased | 0.0146 |
B
| HER-2+ | LvM relative to BrT | p-value | NLvM relative to BrT | p-value | AxM relative to BrT | p-value | NLvM relative to LvM | p-value |
| --- | --- | --- | --- | --- | --- | --- | --- | --- |
| M1 Macrophages | | NS | Decreased | 0.0027 | Increased | 0.0128 | | NS |
| M2 Macrophages | | NS | | NS | | NS | | NS |
| Monocytes | | NS | Decreased | 0.0311 | | NS | | NS |
| Myeloid Dendritic Cells | | NS | | NS | | NS | | NS |
| CD4+ T cells | | NS | | NS | | NS | | NS |
| CD8+ T cells | Decreased | 0.0139 | Decreased | 0.0021 | Increased | 0.0130 | | NS |
| Regulatory T cells | Decreased | <0.0001 | Decreased | 0.0026 | | NS | Increased | 0.0411 |
| B cells | Decreased | 0.0005 | Decreased | 0.0323 | Increased | 0.0281 | Increased | 0.0457 |
| Neutrophils | | NS | | NS | Decreased | 0.0175 | | NS |
| NK cells | | NS | | NS | | NS | | NS |
C
| TNBC | LvM relative to BrT | p-value | NLvM relative to BrT | p-value | AxM relative to BrT | p-value | NLvM relative to BrT | p-value |
| --- | --- | --- | --- | --- | --- | --- | --- | --- |
| M1 Macrophages | | NS | | NS | | NS | Decreased | 0.0115 |
| M2 Macrophages | | NS | | NS | | NS | | NS |
| Monocytes | | NS | | NS | | NS | Increased | 0.0461 |
| Myeloid Dendritic Cells | | NS | Decreased | 0.0466 | | NS | Decreased | 0.0464 |
| CD4+ T cells | Decreased | 0.0271 | | NS | Increased | <0.0001 | | NS |
| CD8+ T cells | Decreased | <0.0001 | | NS | Increased | <0.0001 | Increased | <0.0001 |
| Regulatory T cells | Decreased | 0.0011 | | NS | Increased | <0.0001 | Increased | 0.0004 |
| B cells | Decreased | 0.0372 | | NS | Increased | <0.0001 | Increased | 0.018 |
| Neutrophils | | NS | | NS | Decreased | 0.0063 | | NS |
| NK cells | Increased | 0.0235 | Decreased | 0.0053 | Decreased | 0.0064 | Decreased | 0.0005 |

## Slide 11
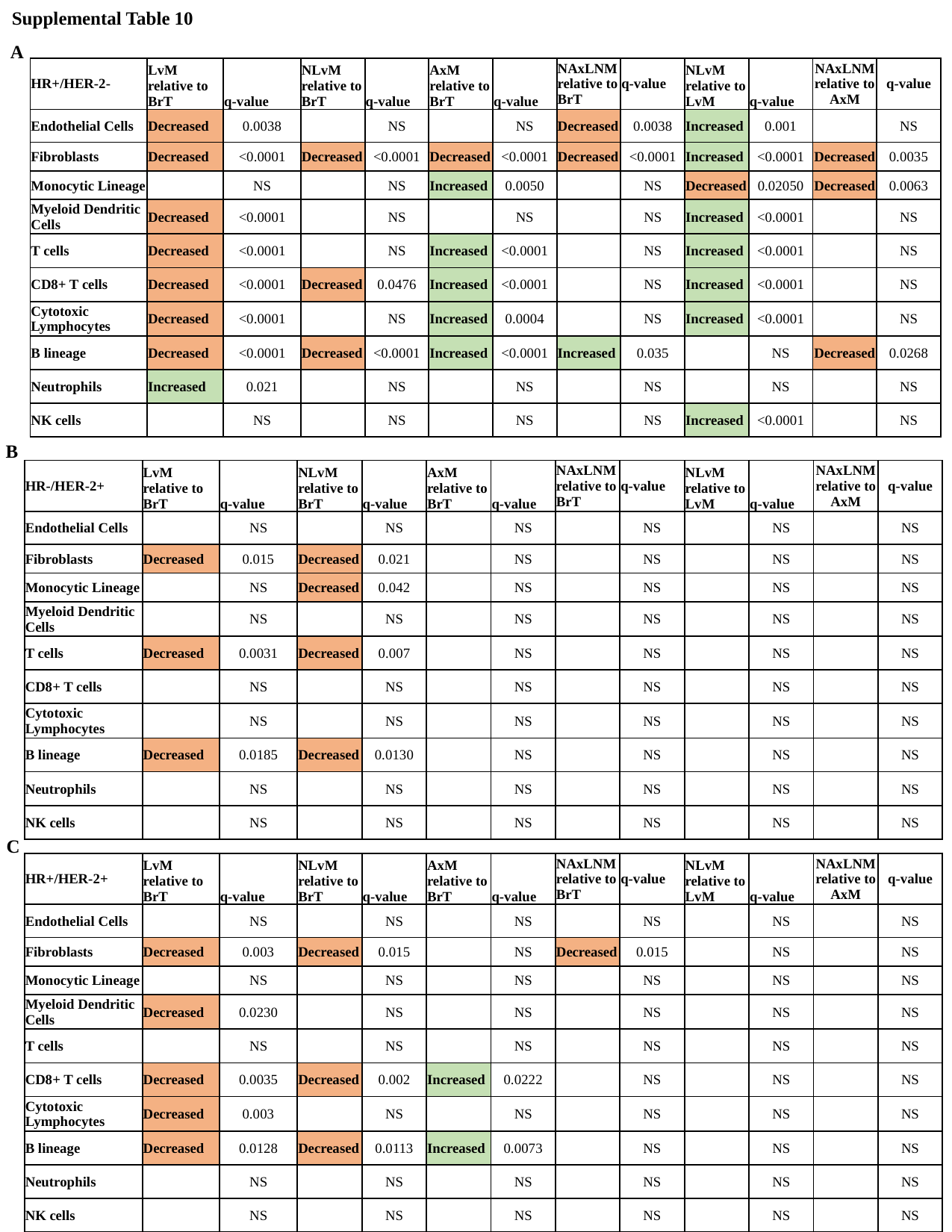

Supplemental Table 10
A
| HR+/HER-2- | LvM relative to BrT | q-value | NLvM relative to BrT | q-value | AxM relative to BrT | q-value | NAxLNM relative to BrT | q-value | NLvM relative to LvM | q-value | NAxLNM relative to AxM | q-value |
| --- | --- | --- | --- | --- | --- | --- | --- | --- | --- | --- | --- | --- |
| Endothelial Cells | Decreased | 0.0038 | | NS | | NS | Decreased | 0.0038 | Increased | 0.001 | | NS |
| Fibroblasts | Decreased | <0.0001 | Decreased | <0.0001 | Decreased | <0.0001 | Decreased | <0.0001 | Increased | <0.0001 | Decreased | 0.0035 |
| Monocytic Lineage | | NS | | NS | Increased | 0.0050 | | NS | Decreased | 0.02050 | Decreased | 0.0063 |
| Myeloid Dendritic Cells | Decreased | <0.0001 | | NS | | NS | | NS | Increased | <0.0001 | | NS |
| T cells | Decreased | <0.0001 | | NS | Increased | <0.0001 | | NS | Increased | <0.0001 | | NS |
| CD8+ T cells | Decreased | <0.0001 | Decreased | 0.0476 | Increased | <0.0001 | | NS | Increased | <0.0001 | | NS |
| Cytotoxic Lymphocytes | Decreased | <0.0001 | | NS | Increased | 0.0004 | | NS | Increased | <0.0001 | | NS |
| B lineage | Decreased | <0.0001 | Decreased | <0.0001 | Increased | <0.0001 | Increased | 0.035 | | NS | Decreased | 0.0268 |
| Neutrophils | Increased | 0.021 | | NS | | NS | | NS | | NS | | NS |
| NK cells | | NS | | NS | | NS | | NS | Increased | <0.0001 | | NS |
B
| HR-/HER-2+ | LvM relative to BrT | q-value | NLvM relative to BrT | q-value | AxM relative to BrT | q-value | NAxLNM relative to BrT | q-value | NLvM relative to LvM | q-value | NAxLNM relative to AxM | q-value |
| --- | --- | --- | --- | --- | --- | --- | --- | --- | --- | --- | --- | --- |
| Endothelial Cells | | NS | | NS | | NS | | NS | | NS | | NS |
| Fibroblasts | Decreased | 0.015 | Decreased | 0.021 | | NS | | NS | | NS | | NS |
| Monocytic Lineage | | NS | Decreased | 0.042 | | NS | | NS | | NS | | NS |
| Myeloid Dendritic Cells | | NS | | NS | | NS | | NS | | NS | | NS |
| T cells | Decreased | 0.0031 | Decreased | 0.007 | | NS | | NS | | NS | | NS |
| CD8+ T cells | | NS | | NS | | NS | | NS | | NS | | NS |
| Cytotoxic Lymphocytes | | NS | | NS | | NS | | NS | | NS | | NS |
| B lineage | Decreased | 0.0185 | Decreased | 0.0130 | | NS | | NS | | NS | | NS |
| Neutrophils | | NS | | NS | | NS | | NS | | NS | | NS |
| NK cells | | NS | | NS | | NS | | NS | | NS | | NS |
C
| HR+/HER-2+ | LvM relative to BrT | q-value | NLvM relative to BrT | q-value | AxM relative to BrT | q-value | NAxLNM relative to BrT | q-value | NLvM relative to LvM | q-value | NAxLNM relative to AxM | q-value |
| --- | --- | --- | --- | --- | --- | --- | --- | --- | --- | --- | --- | --- |
| Endothelial Cells | | NS | | NS | | NS | | NS | | NS | | NS |
| Fibroblasts | Decreased | 0.003 | Decreased | 0.015 | | NS | Decreased | 0.015 | | NS | | NS |
| Monocytic Lineage | | NS | | NS | | NS | | NS | | NS | | NS |
| Myeloid Dendritic Cells | Decreased | 0.0230 | | NS | | NS | | NS | | NS | | NS |
| T cells | | NS | | NS | | NS | | NS | | NS | | NS |
| CD8+ T cells | Decreased | 0.0035 | Decreased | 0.002 | Increased | 0.0222 | | NS | | NS | | NS |
| Cytotoxic Lymphocytes | Decreased | 0.003 | | NS | | NS | | NS | | NS | | NS |
| B lineage | Decreased | 0.0128 | Decreased | 0.0113 | Increased | 0.0073 | | NS | | NS | | NS |
| Neutrophils | | NS | | NS | | NS | | NS | | NS | | NS |
| NK cells | | NS | | NS | | NS | | NS | | NS | | NS |

## Slide 12
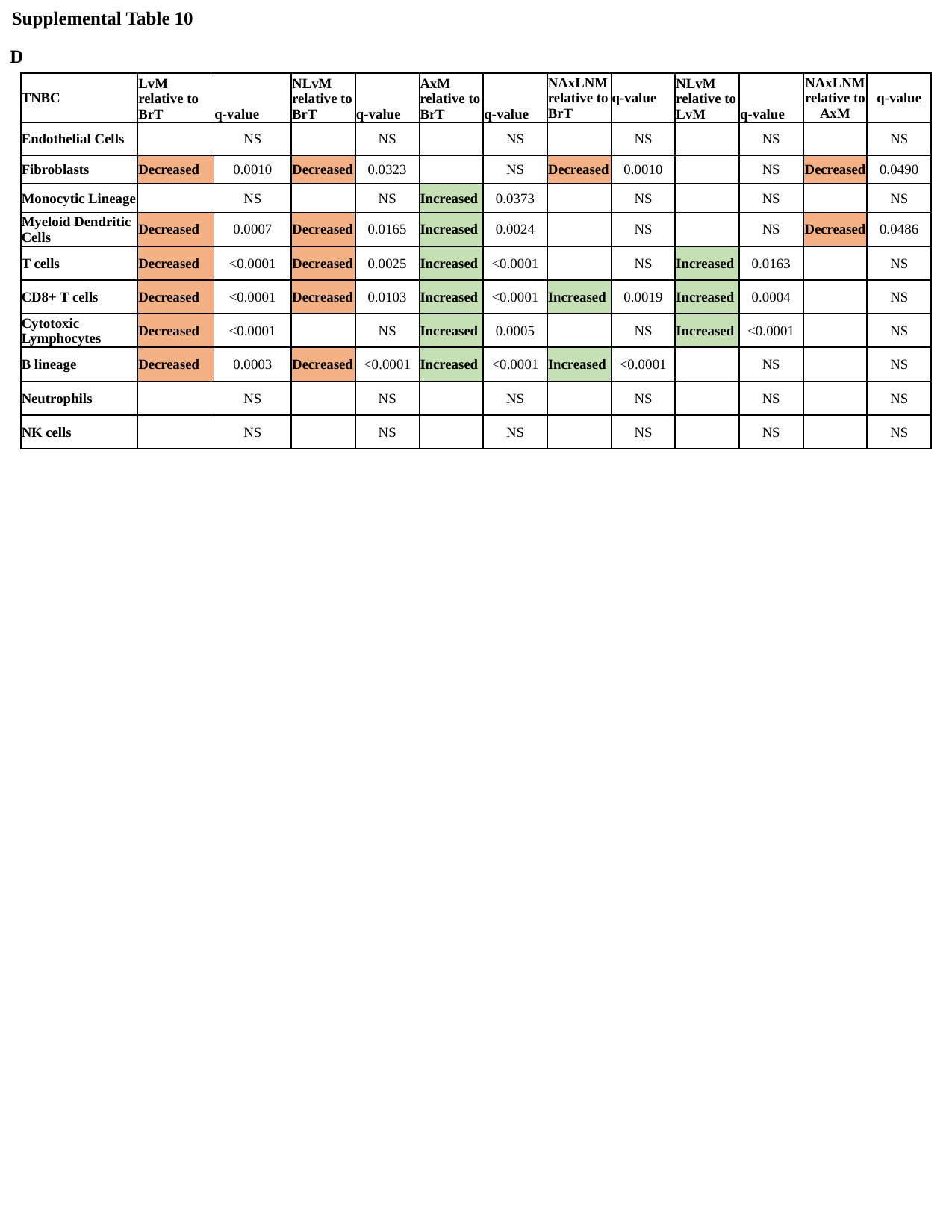

Supplemental Table 10
D
| TNBC | LvM relative to BrT | q-value | NLvM relative to BrT | q-value | AxM relative to BrT | q-value | NAxLNM relative to BrT | q-value | NLvM relative to LvM | q-value | NAxLNM relative to AxM | q-value |
| --- | --- | --- | --- | --- | --- | --- | --- | --- | --- | --- | --- | --- |
| Endothelial Cells | | NS | | NS | | NS | | NS | | NS | | NS |
| Fibroblasts | Decreased | 0.0010 | Decreased | 0.0323 | | NS | Decreased | 0.0010 | | NS | Decreased | 0.0490 |
| Monocytic Lineage | | NS | | NS | Increased | 0.0373 | | NS | | NS | | NS |
| Myeloid Dendritic Cells | Decreased | 0.0007 | Decreased | 0.0165 | Increased | 0.0024 | | NS | | NS | Decreased | 0.0486 |
| T cells | Decreased | <0.0001 | Decreased | 0.0025 | Increased | <0.0001 | | NS | Increased | 0.0163 | | NS |
| CD8+ T cells | Decreased | <0.0001 | Decreased | 0.0103 | Increased | <0.0001 | Increased | 0.0019 | Increased | 0.0004 | | NS |
| Cytotoxic Lymphocytes | Decreased | <0.0001 | | NS | Increased | 0.0005 | | NS | Increased | <0.0001 | | NS |
| B lineage | Decreased | 0.0003 | Decreased | <0.0001 | Increased | <0.0001 | Increased | <0.0001 | | NS | | NS |
| Neutrophils | | NS | | NS | | NS | | NS | | NS | | NS |
| NK cells | | NS | | NS | | NS | | NS | | NS | | NS |

## Slide 13
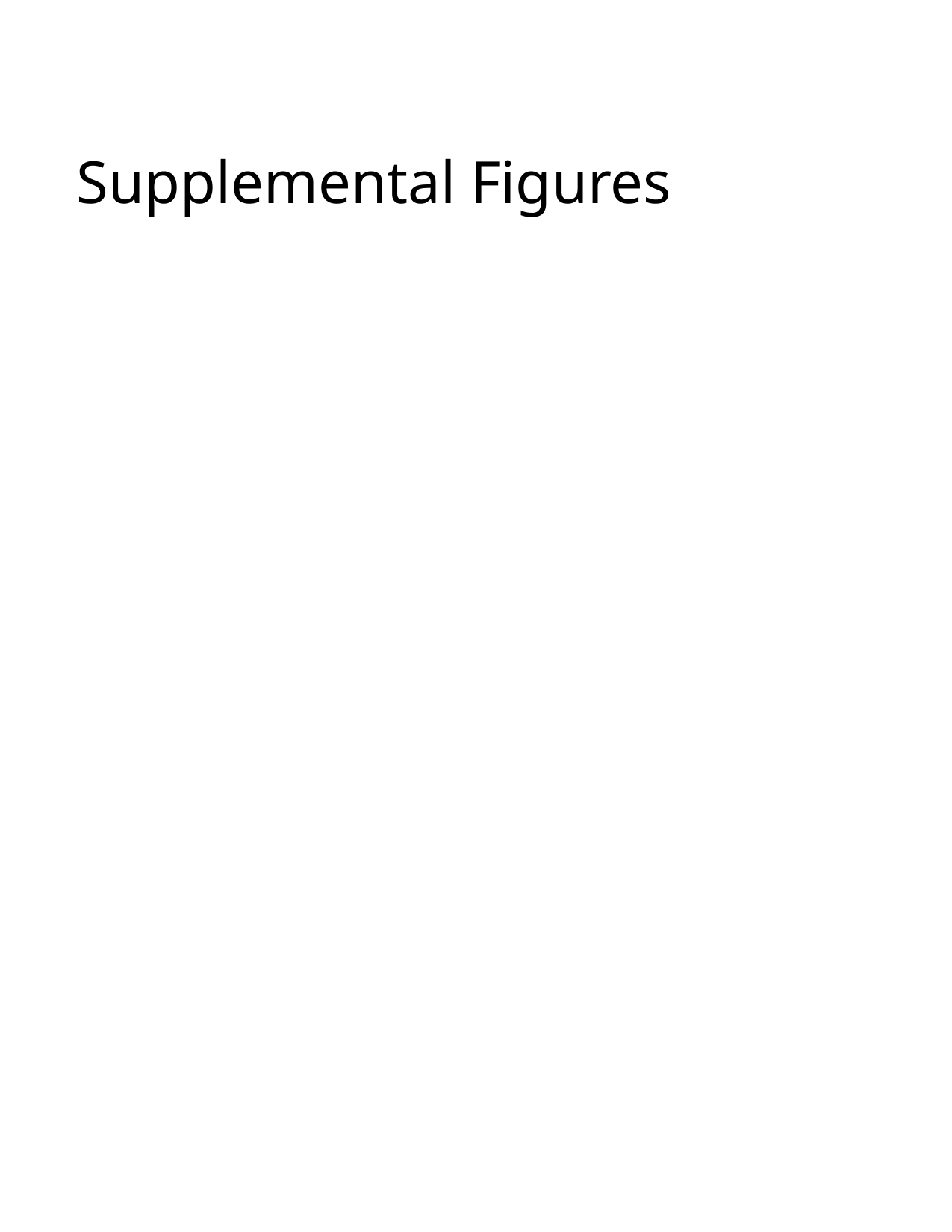

# Supplemental Figures

## Slide 14
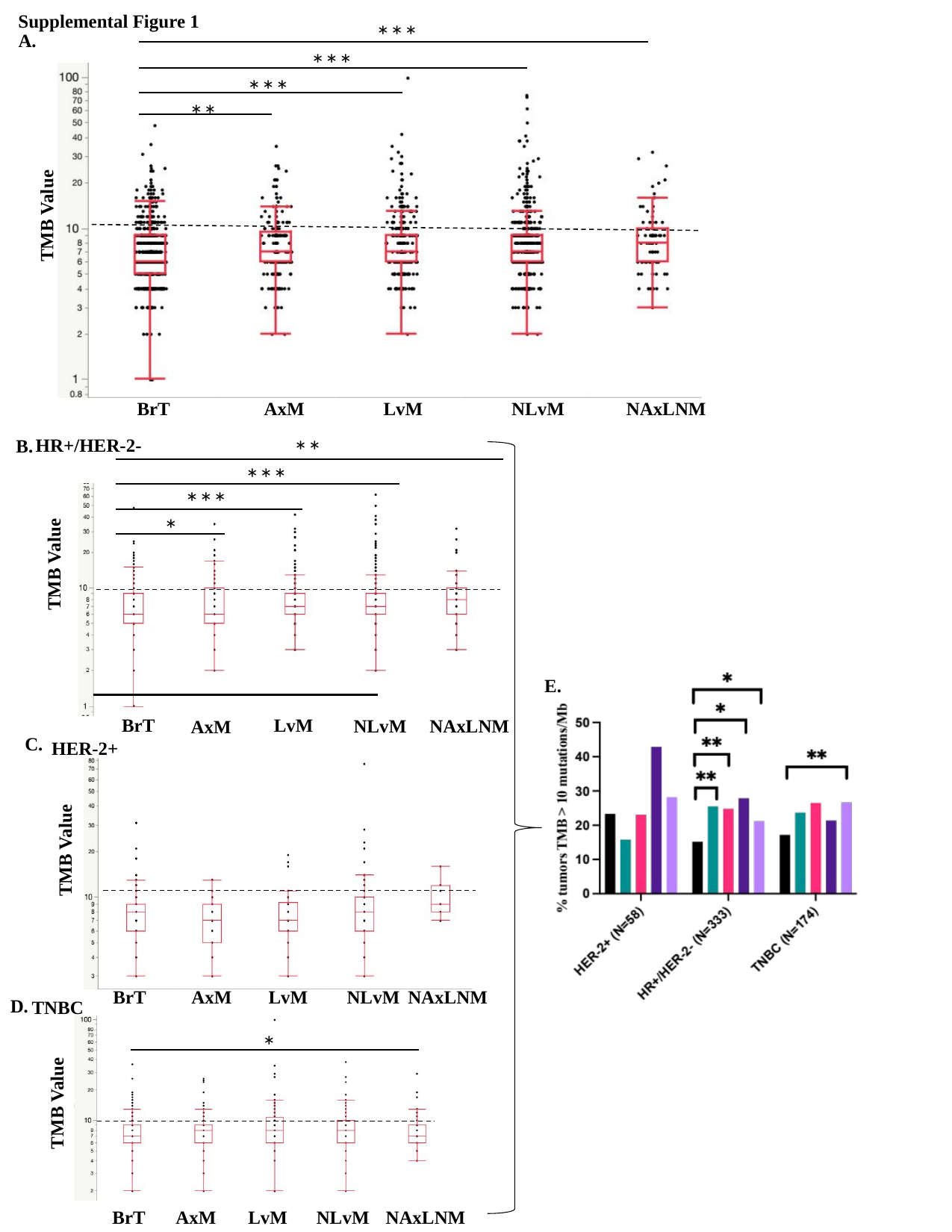

# Supplemental Figure 1
***
A.
***
***
**
TMB Value
BrT
AxM
LvM
NLvM
NAxLNM
**
HR+/HER-2-
B.
***
***
*
TMB Value
BrT
LvM
NLvM
NAxLNM
AxM
E.
C.
HER-2+
TMB Value
BrT
AxM
LvM
NLvM
NAxLNM
D.
TNBC
*
TMB Value
BrT
AxM
LvM
NLvM
NAxLNM
AxM

## Slide 15
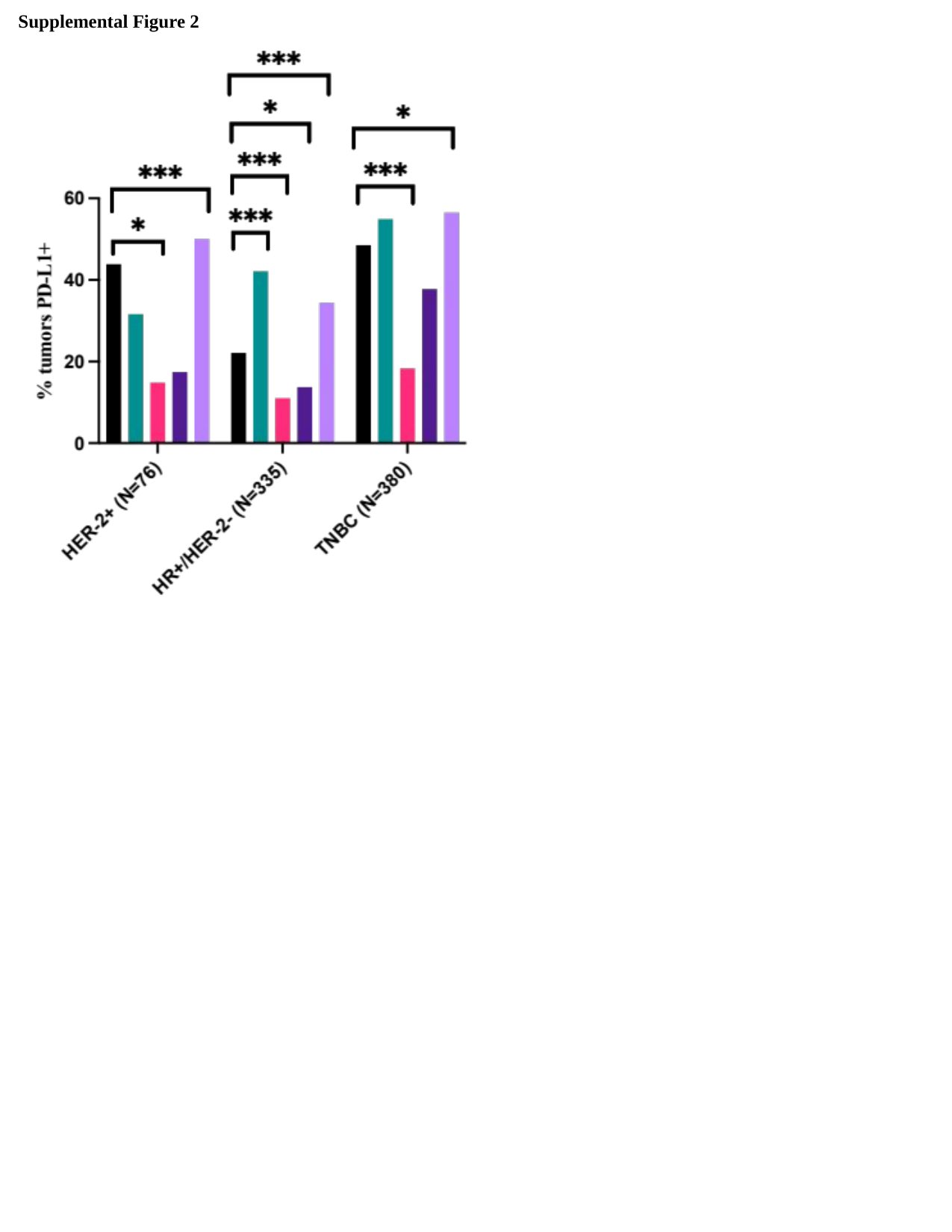

# Supplemental Figure 2
